# When Ontogeny Matters: A New Japanese Species of Brittle Star Illustrates the Importance of Considering both Adult and Juvenile Characters in Taxonomic Practice

**DOI:** 10.1371/journal.pone.0139463

**Published:** 2015-10-28

**Authors:** Alexander Martynov, Yoshiaki Ishida, Seiichi Irimura, Rie Tajiri, Timothy O’Hara, Toshihiko Fujita

**Affiliations:** 1 Zoological Museum, Moscow State University, Moscow, Russia; 2 National Museum of Nature and Science, Tsukuba, Japan; 3 Department of Earth Science, Waseda University, Tokyo, Japan; 4 Museum Victoria, Melbourne, Victoria, Australia; Laboratoire de Biologie du Développement de Villefranche-sur-Mer, FRANCE

## Abstract

Current taxonomy offers numerous approaches and methods for species delimitation and description. However, most of them are based on the adult characters and rarely suggest a dynamic representation of developmental transformations of taxonomically important features. Here we show how the underestimation of ontogenetic changes may result in long term lack of recognition of a new species of one of the most common ophiacanthid brittle stars (Echinodermata: Ophiuroidea) from the North Pacific. Based on vast material collected predominantly by various Japanese expeditions in the course of more than 50 years, and thorough study of appropriate type material, we revise the complex of three common species of the ophiuroid genus *Ophiacantha* which have been persistently confused with each other. The present study thus reveals the previously unrecognized new species *Ophiacantha kokusai* sp.nov. which is commonly distributed off the Pacific coast of Japan. The new species shows developmental differentiation from the closely related species *Ophiacantha rhachophora* H. L. Clark, 1911 and retains clearly expressed early juvenile features in the adult morphology. Another species, *Ophiacantha clypeata* Kyte, 1977, which had been separated from *O*. *rhachophora*, is in turn shown to be just a juvenile stage of another North Pacific species, *Ophiacantha trachybactra* H.L. Clark, 1911. For every species, detailed morphological data from both adult and juvenile specimens based on scanning electron microscopy are presented. A special grinding method showing complex internal features has been utilized for the first time. For all three species in this complex, a clear bathymetric differentiation is revealed: *O*. *rhachophora* predominantly inhabits shallow waters, 0–250 m, the new species *O*. *kokusai* lives deeper, at 250–600 m, and the third species, *O*. *trachybactra*, is found at 500–2,000 m. The present case clearly highlights the importance of considering developmental transformations, not only for a limited number of model organisms, but as part of the taxonomic process.

## Introduction

Taxonomy is a core discipline of biology [1], although for a long time it has been a rather neglected subject [2,3]. Because of the complexity of hierarchical levels of biological patterns and processes, many methods for organism classification have been proposed [4–9], including recent integrative approaches [10]. The general importance of ontogenetic data has been acknowledged since Haeckel [11], Garstang [12] and Gould’s [13] seminal works, as well as recent evo-devo achievements [14–18]. However, apart from a few studies [19, 20], current taxonomy and phylogenetic inference generally remains almost “development-free” and principally focuses on the adult stages. For example, the number of publications on the ISI Web of Knowledge (for period 1865–2015, on 12 February 2015) with key terms describing the interaction between evolution and ontogeny (heterochrony and paedomorphosis [21]) are still very small. For example, a search performed on the largest phylum of all living organisms, Arthropoda, arthropod*+heterochron(y)* resulted in just 152 publications, whereas arthropod*+taxonom(y)* resulted in 121,988 publications. A similar search for the phylum Echinodermata gave 88 and 2,465 publications respectively. Even the widest scope (e.g., arthropod*+taxonom(y)*+development* = 6,732; echinoderm*+taxonom(y)*+development* = 260) delivers noticeably fewer than “taxonomy” alone (i.e. 121988 and 2465 respectively) and contains numerous off topic citations. The term “taxonomy” is absent from recent schemes explaining the evo-devo synthesis [22]. Attempts to employ development in taxonomy and phylogenetics are thus currently exceptions rather than common practice. Evolution is normally represented in the form of a branched tree and does not necessarily encompass the complex ontogenetic cycle, although the importance of the ‘evolution of development’ has been highlighted [23].

Here we therefore present the discovery of a new abundant species of brittle star within a very intensively studied area, that has remained unrecognized, and confused with close relatives, precisely because ontogenetic heterochronic shifts in several important characters were previously underestimated. Importantly, because collections of marine invertebrates are heavily dependent on complicated and expensive vessel expeditions which normally do not target specific taxonomic projects, the available material is either old or fixed in formalin (like many specimens used for this study) that prevent a proper molecular study. The ontogenetic presentation of taxonomic characters, as well as being of general importance for evo-devo studies, helps to reveal new “cryptic” species using morphological data.

Brittle-stars (ophiuroids) are a remarkable group of marine invertebrates of the phylum Echinodermata. Ophiuroids play an important role in a variety of ecosystems, and they are dominant organisms of bottom communities from the intertidal to the deepest marine trenches at 5,000–7,000 m [24]. They can also display an array of unusual anatomical systems, such as a unique vision system using skeletal elements of the arms as an optical instrument [25]. The taxonomy of the Ophiacanthidae is one of the most problematic among brittle stars [26–31]. Numerous genera with uncertain diagnoses and absence of a robust phylogeny are among major challenges to ophiacanthid studies. New ophacanthid species continue to be described [28, 30, 31], however they are usually discovered in low numbers within a restricted locality. Here we present a detailed morphological description of a common new species of the genus *Ophiacantha* from the Pacific side of the Japanese Islands in an ontogenetic framework and provide comparison with two closely related species. The present study is based on abundant material which was collected over 40 years by numerous expeditions around all main Japanese Islands, from Okinawa and the East China Sea regions, to Northern Honshū [32–36]. The type material for the newly described species exceeds 2,000 specimens.

## Material and Methods

Samples were obtained during cruises of several research vessels from 1963 to 2005 around Japan and are now deposited in the National Museum of Nature and Science, Tsukuba (NSMT). Type and other additional specimens from the National Museum of Natural History, Smithsonian Institution, Washington DC (USNM), Museum of Comparative Zoology, Harvard University (MCZ), Muséum National d’Histoire Naturelle, Paris (MNHN), Natural History Museum, London (BMNH), Zoological Museum, Danish Museum of Natural History (ZMUC), Copenhagen, Zoological Museum of Moscow State University (ZMMU), and Zoological Institute, St. Petersburg (ZIN RAS), were also examined. Skeletal elements were isolated by bleaching in domestic bleach (NaOCl), rinsed in water and dried. Postlarval ophiuroid specimens were obtained from the same sample as adults, or from a location in close proximity. The identification of small juveniles was based on the study of a growth series, from adult stages with definite specific characters backwards to the earlier postlarval stages where the number of diagnostic characters diminishes [37–39]. Scanning electron microscopy (SEM) of coated mounts of both adults and juveniles was performed with a JSM–6380 and of uncoated specimens with a Keyence VHX-D510 in National Museum of Nature and Science, Tsukuba, Japan.

A recently developed method to observe animal internal tissues and hard structures using resin impregnation and geological grinding [40] was applied here to ophiuroid taxonomy for the first time to confirm the exact position of key skeletal elements. The method included the following steps. 1) Dehydration by acetone (5 gradations 80 to 99.8%). 2) Immersion in Spurr resin. 3) Replacement of acetone with resin by decompression (the specimen in resin was put in a decompression device, the pressure was slowly decreased to 10 kPa, after 12 hours, the resin was replaced). 4) Polymerization by heating (after repeating the above process 5 times, the specimen immersed with resin was warmed at 60°C for 48 hours to cause thermal polymerization; after 48 hours, the completely solidified specimen was cooled down at room temperature). 5) Cutting by a rock cutter. 6) Grinding (the surface was polished with a carbon abrasive (#800) by using a grinder, with almina abrasive (#1500) on a glass plate, and with almina abrasive (#3000) and water on the glass plate) and simultaneous observation by reflected light. A continuous section of a specimen can be created by repeated polishing and photography. 7) Adhering the sample to a glass slide. 8) Grinding the opposite surface. 9) Observation by transmitted light.

Seafloor temperature data were obtained from the Japan Oceanographic Data Center portal (http://www.jodc.go.jp/index.html). Bathymetric and temperature data were evaluated statistically using nonparametric Mann-Whitney rank sum tests (SigmaPlot for Windows Version 11.0).

### Nomenclatural Acts

The electronic edition of this article conforms to the requirements of the amended International Code of Zoological Nomenclature, and hence the new names contained herein are available under that Code from the electronic edition of this article. This published work and the nomenclatural acts it contains have been registered in ZooBank, the online registration system for the ICZN. The ZooBank LSIDs (Life Science Identifiers) can be resolved and the associated information viewed through any standard web browser by appending the LSID to the prefix "http://zoobank.org/". The LSID for this publication is: urn:lsid:zoobank.org:pub: 1DE3E2C5-8722-42B0-97B1-9A9AB11252A6. The electronic edition of this work was published in a journal with an ISSN, and has been archived and is available from the following digital repositories: PubMed Central, LOCKSS.

### Major features of ophiuroid postlarval development

Ophiuroids display remarkable changes during postlarval and early juvenile development. Brittle star postlarval ontogeny is relatively well studied compared to other invertebrates due to the availability of growth series from seafloor samples. To date, various data have been accumulated on postlarval development for the majority of ophiuroid families [24, 37–39, 41–45]. However, a unified model of brittle-star postlarval development has not been suggested. Such a model would be useful as a practical tool for understanding the ophuroid phylogeny and resolving complicated taxonomic problems. Here we outline a scheme for ophiuroid postlarval development (based on published data [24, 37–39, 41–45] and this study (new data on the postlarval development of several species from diverse ophiuroid families, such as Ophiacanthidae, Ophiuridae and Amphiuridae)) that will be used as a framework to describe the ontogenetic development of three species of *Ophiacantha*.

The main changes in ophiuroid skeleton formation occur between stages with disk diameter ca. 0.3–2 mm. The ophiuroid postlarvae have a conserved morphology as follows:

1. The major part of the dorsal disk is occupied by the primary plate rosette comprised of a single central primary plate and usually 5 radial primary plates.
2. The radial shields and genital plates have yet to appear or are underdeveloped.
3. Each half-jaw is narrow, elongate and bears ventrally a few rudimentary oral papillae, commonly bar-shaped.
4. The dental plate is small, convex and bears a few tooth sockets of unspecific shape.
5. Each adoral shield bears a papilla (spine) of various length and shape.
6. Arm segments are limited in number and considerably elongated proximally.
7. Vertebrae are comprised of two separate, loosely connected elongated parts.
8. The vertebral articulation is generally underdeveloped without a well-defined condyle distally.

Ophiuroids may reach reproductive maturity at 3–5 mm disk diameter, but more often the adult state is attained at a larger size, ca. >10–30 mm disk diameter. However, despite a diversity of adult features, there are common patterns which correspond to the postlarval characters listed above:

1. The major part of the dorsal disk is typically occupied by numerous small scales. The primary rosette, if present, occupies only a small area in the central part of the disk.
2. Radial shields and genital plates are fully developed and well defined, forming a characteristic articulation with each other and supporting the genital slits.
3. Half-jaws develop into varying shapes but are commonly quite high and bear several distinct oral papillae, often numerous, spiniform or leaf-like.
4. Dental plate is large, flattened and bears numerous tooth sockets of specific shape.
5. Adoral shield papilla (spine) may be retained in the adult state or migrated to the jaws to become one of the distal oral papillae.
6. Arm segments are generally numerous and can be considerably elongated distally.
7. Vertebrae are entire, the former halves are now tightly fused; only indistinct sutures remain.
8. Vertebral articulation is fully developed. Zygospondylous articulations, with a well-defined distal condyle, occur in the majority of families; in others streptospondylous articulations appear.

In a particular species, characters may represent a mixture of strictly postlarval and adult features [46–49], due to heterochronies and other developmental alterations [21, 50, 51] that link ontogeny, as processes of the individual development, and evolution, as processes of historical transformations of organisms [52]. In extreme cases postlarval characters can dominate in the adult organism thus representing the process of paedomorphosis in a narrow sense [21]. The latter has been discovered in many metazoan phyla, including such large and ecologically important groups as annelids, mollusks, echinoderms and also in flowering plants [46, 53–55]. Adult stages thus may demonstrate not only an “overall paedomorphosis”, but some particular postlarval features, which can be easily overlooked without having an appropriate model of the ontogenetic development of the group. A striking example of the underestimation of ontogenetic data is discovered in this study.

## Results

### Taxonomy

*Ophiacantha kokusai* sp. nov. (Figs 1–8)

**Fig 1:**
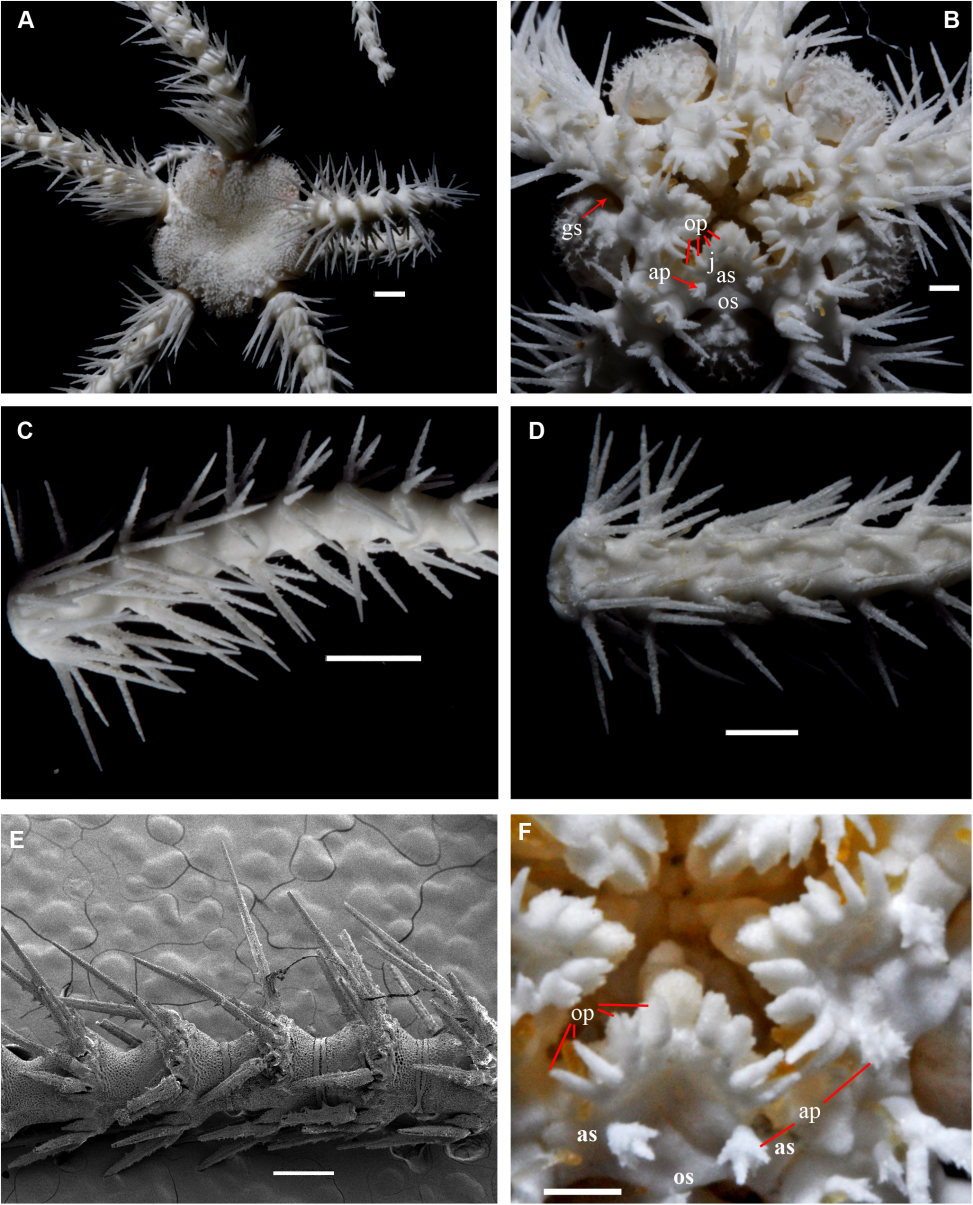
*Ophiacantha kokusai* sp.nov., holotype NSMT E–3188 from Owase Trough, Kii Peninsula, 5.1 mm dd, external views. **A,** dorsal view; **B,** ventral view; **C,** proximal arm segments, dorsal view; **D,** proximal arm segments, ventral view; **E,** proximal arm segments, lateral view, SEM; **F,** oral frame, details. ap, adoral shield papilla; as, adoral shields; gs, genital slit; j, jaws; op, oral papillae; os, oral shield. Scale bars, 0.5 mm (E), 1 mm (A, C, D), 0.2 mm (B, F).

**Fig 2:**
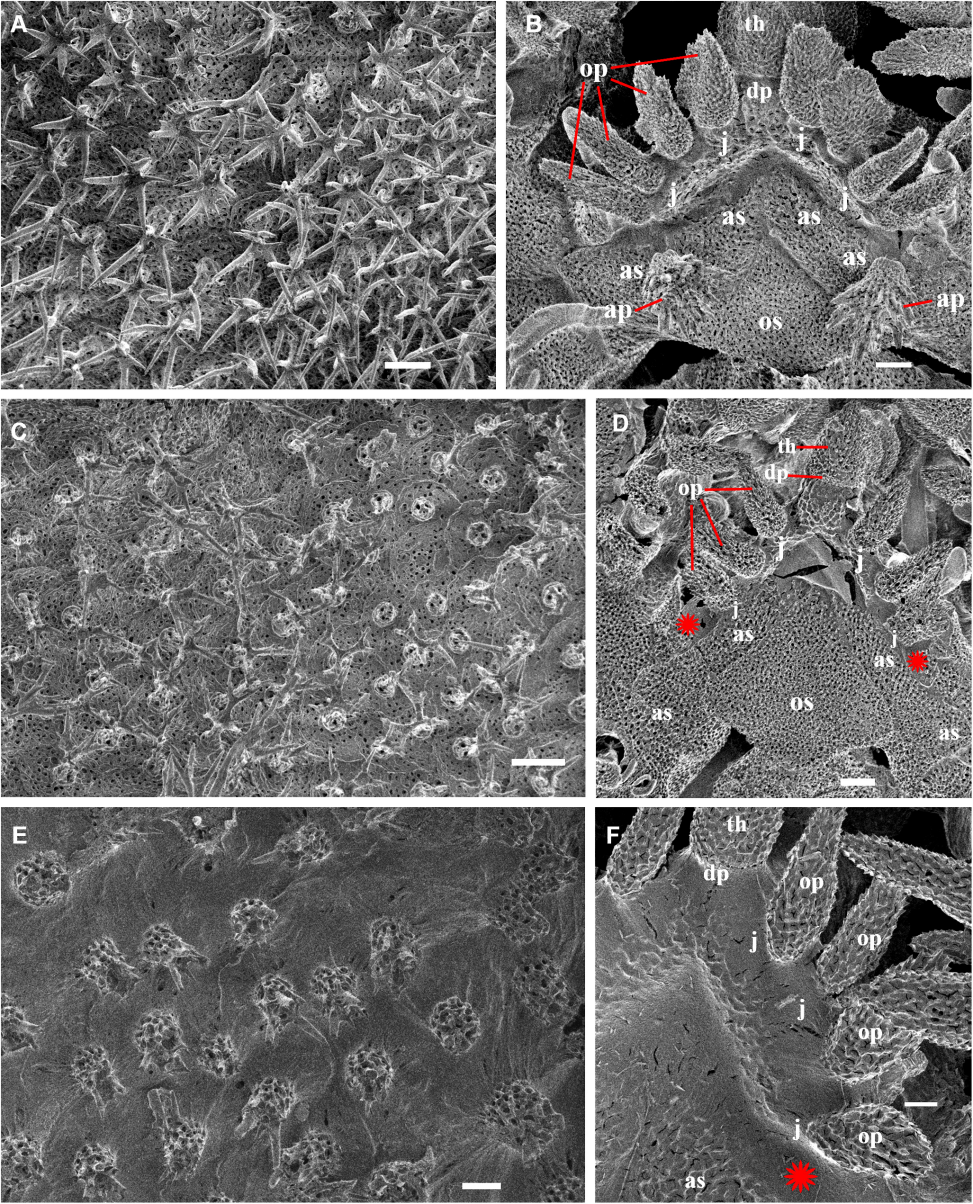
Adult disk spines and oral frames of *Ophiacantha kokusai* sp.nov. (A–B, holotype), *Ophiacantha rhachophora* H.L. Clark, 1911 (C–D, NSMT E–1540) and *Ophiacantha trachybactra* H.L. Clark, 1911 (E–F, NSMT E–7543). **SEM of uncoated specimens using Keyence VHX-D510**. ap, adoral shield papillae; as, adoral shields; dp, dental plate; gs, genital slit; j, jaws; op, oral papillae; os, oral shield; th, teeth; red asterisks indicate absence of the adoral shield papillae in adult *O*. *rhachophora* and *O*. *trachybactra*. Scales bars, 0.1 mm.

**Fig 3:**
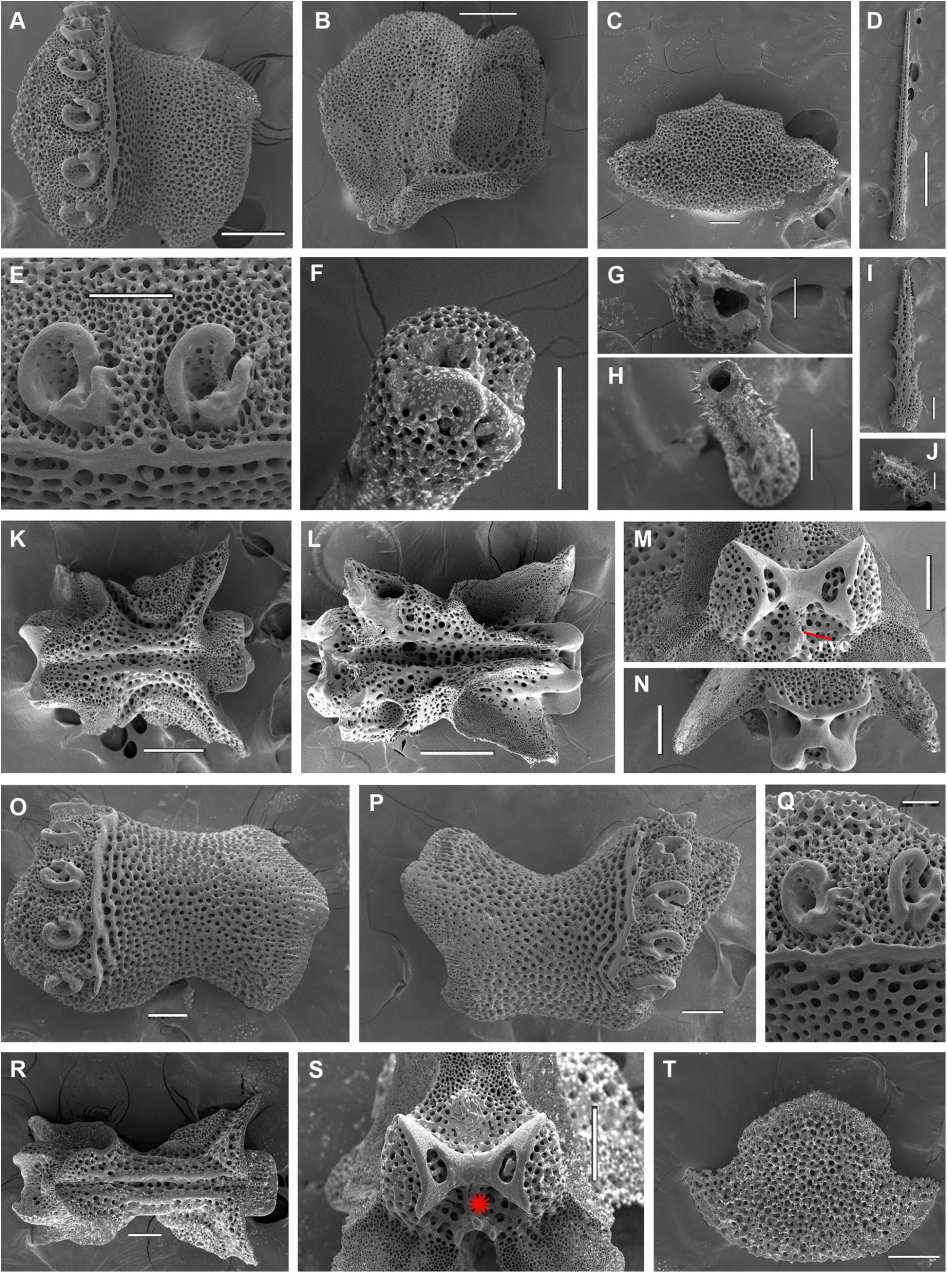
*Ophiacantha kokusai* sp.nov., holotype NSMT E–3188, details, SEM. A–N, proximal segments. **A,** lateral arm plate; **B,** same, inside view; **C,** ventral arm plate; **D,** dorsal spine; **E,** arm spine articulations; **F,** spine, ventral view; **G,** hollow dorsal spine, transversally sectioned at basal part; **H,** hollow dorsal spine, mid transverse section; **I,** ventral spine; **J,** ventral spine mid-section showing small cavities; **K,** vertebra, dorsal view; **L,** same, ventral view; **M,** same, distal view; **N,** same, proximal view; **O–T, middle segments. O–P,** lateral arm plates showing no protuberances; **Q,** arm spine articulations; **R,** vertebra, dorsal view; **S,** same, distal view; **T,** ventral arm plate. vc, vertebral condyle on proximal vertebrae; red asterisk indicates the absence of vertebral condyle on more distal vertebrae. Scales bars, 0.05 (G, J, Q), 0.1 mm (C, E, F, H, I, M–O, P–T), 0.2 mm (A, B, K, L), 0.5 mm (D).

**Fig 4:**
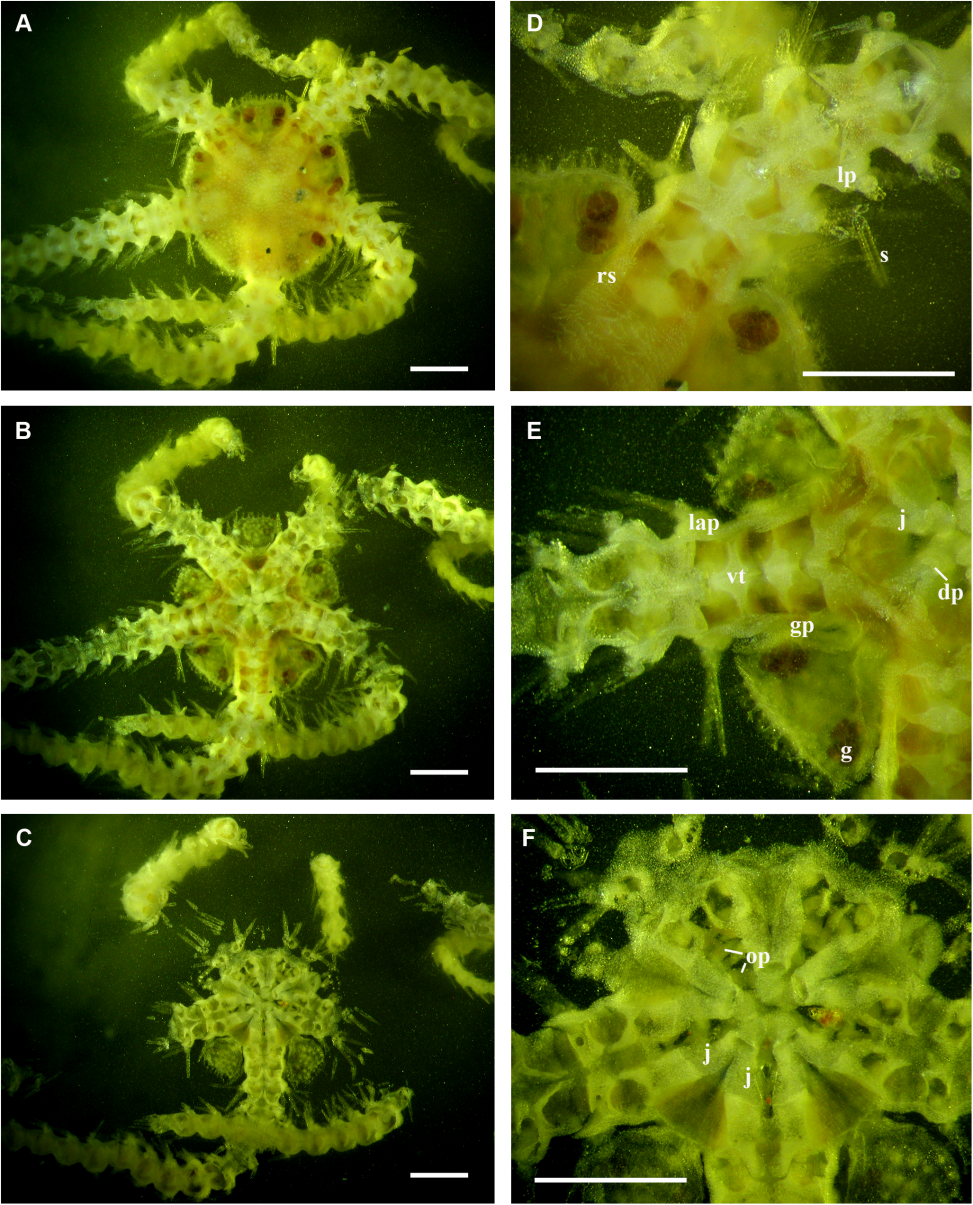
*Ophiacantha kokusai* sp.nov., paratype NSMT E–7638 from Sagami Bay, internal structures of entire specimen uncovered using the grinding method. **A,D,** dorsalmost sections; **B,E,** middle sections; **C,F,** ventralmost sections. dp, dental plate; g, gonads; gp, genital plates; j, jaws; lap, lateral arm plates; op, oral papillae; rs, radial shields; s, spines; vt, vertebrae. Scale bars, 1 mm.

**Fig 5:**
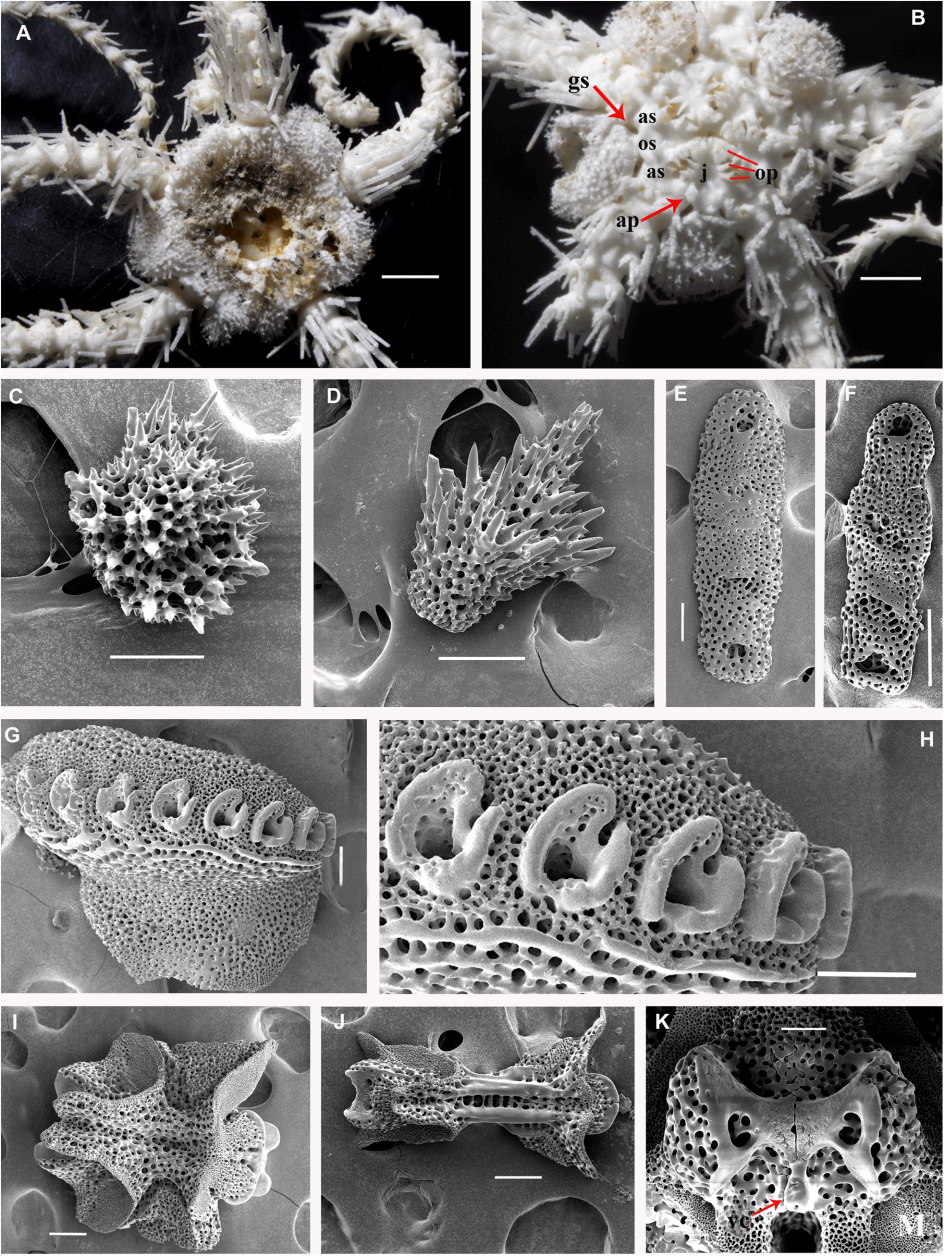
*Ophiacantha kokusai* sp.nov., paratype NSMT E–7596 from Sagami Bay, 4.5 mm dd, external views and details, SEM. **A,** dorsal view; **B,** ventral view; **C,D,** thorny adoral shield papilla with central spine and numerous fine thorns; **E,** dental plates; **F,** dorsal spine; **G,** third lateral arm plate (first free proximal segment) closely adjacent to the disk showing striated elevation near articulations; **H,** arm spine articulations; **I,** proximal vertebra, dorsal view; **J,** middle vertebra, dorsal view; **K,** proximal vertebra, distal view; Scales bars, 0.05 (I), 0.1 mm (C, D, E, F, G, H, J, K), 1 mm (A, B). ap, adoral shield papilla; as, adoral shields; gs, genital slit; op, oral papillae; os, oral shield; vc, vertebral condyle of proximal vertebrae (zygospondylous articulation).

**Fig 6:**
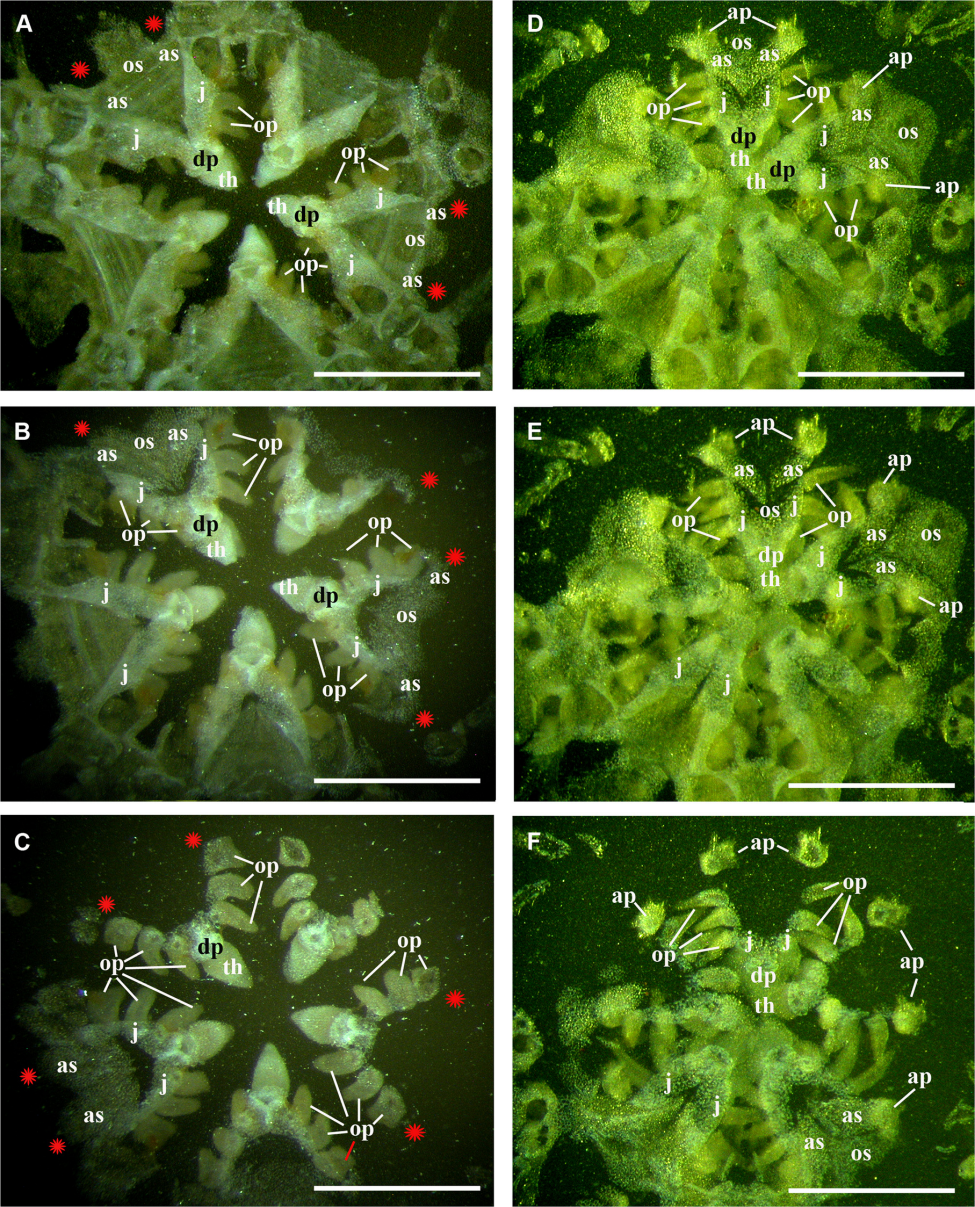
Comparison of the oral frame patterns in *O*. *rhachophora* H.L. Clark 1911 and *O*. *kokusai* sp. nov. and (specimen NSMT E–1540 and paratype NSMT E–7638 respectively) using the grinding method technique. **A–C,** three consecutive sections of the disk of *O*. *rhachophora* showing placement of only three oral papillae (including distalmost one) emerging exclusively from the jaws; **D–F,** three consecutive sections of the disk of ***O*. *kokusai* sp.nov.** showing placement of three oral papillae emerging from jaws and a fourth separate thorny papilla emerging from the adoral shield. ap, adoral shield papillae; as, adoral shields; dp, dental plate; gs, genital slit; j, jaws; op, oral papillae; os, oral shield; th, teeth; red asterisks indicate the absence of the adoral shield papillae in *O*. *rhachophora* (**A–C**). Scale bars, 1 mm.

**Fig 7:**
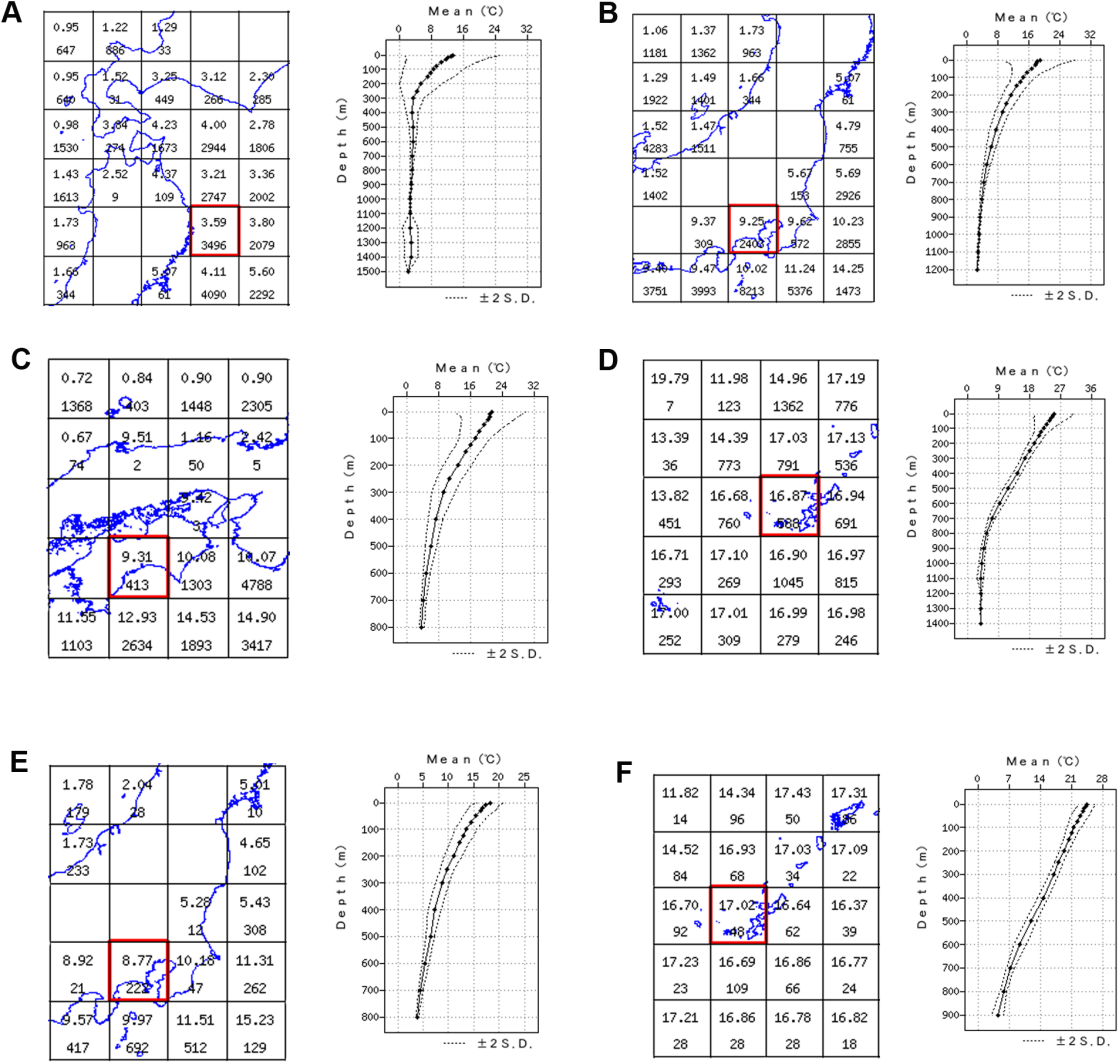
(A–D) Mean annual temperatures for the waters off Northern Honshū and Hokkaido (A), central Honshū (B), Southern Honshū and Shikoku (C), Okinawa (D). On each figure the left image represents mean annual temperature values at the depth of 300 m (upper value in each cell; lower value in each cell–number of years with measurements) and the right image represents annual water temperature at different depth horizons for particular locality (corresponding to particular cells in the red frame). (E) Mean annual temperatures for May off the central Honshū. (F) Mean annual temperatures for May off Okinawa. Same figure designations as for (A–D). Data compiled from the Japan Oceanographic Data Center portal (http://www.jodc.go.jp/index.html).

**Fig 8:**
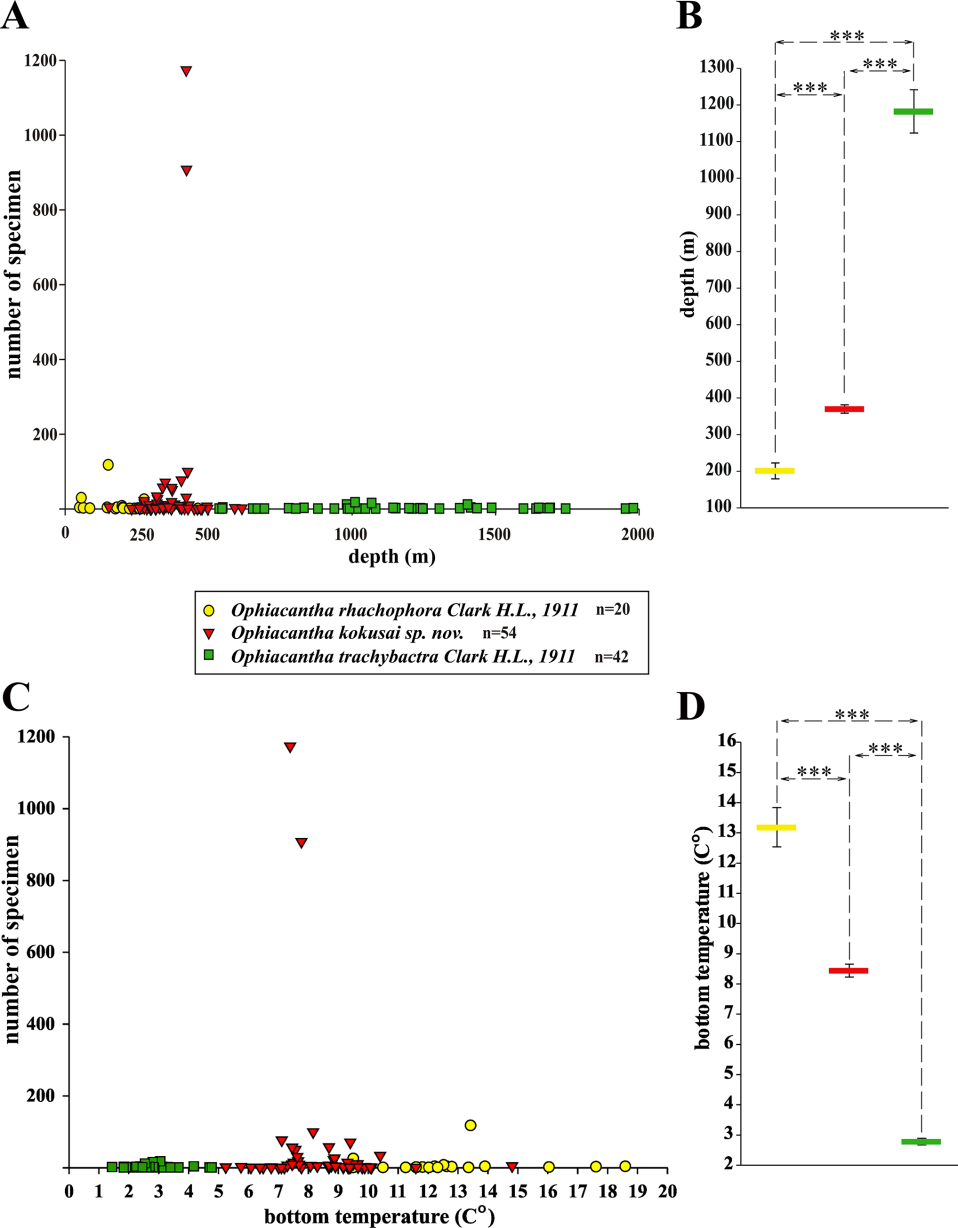
(A) Bathymetric differentiation among a complex of three related species of the brittle-star genus *Ophiacantha* in the Northern Pacific (including new species *O*. *kokusai)*. (В) Mean bathymetric distribution for the three species. (C) Differentiation of thermal tolerance for the three species of the genus *Ophiacantha*. (D) Mean temperature preferences for the three species. Data were evaluated statistically using nonparametric Mann-Whitney rank sum test (SigmaPlot for Windows Version 11.0), differences were considered significant at ***p < 0.001. n–number of samples.

*Ophiacantha rhachophora* auct. non H.L. Clark, 1911 [56] (partim., excluding the holotype and some paratypes)

Urn:lsid:zoobank.org:act: 1F8411A-BCEB-4557-8C24-2BC720A1C78B

#### Etymology

From *kokusai* (Japanese, 国際, こくさい) means “international” in reference to the team of researchers from different countries involved in this project on the Japanese brittle-stars.

#### Holotype

NSMT E–3188, dry, SEM stubs # 40–42, T/S *Seisui-maru*, sand, 06-06-1991, off Owase Trough, Kii Peninsula, Wakayama Pref., depth 378–545 m, basket, collector M. Saba.

#### Paratypes

(2,793 specimens, see S1 Appendix)

#### Description of the adult holotype

The disk is 5.1 mm in diameter (dd), not indented interradially. The disk scales are concealed by thin skin and numerous spines (**Fig 1A**). There are 3–5 primary branches and up to 12 smaller, secondary and tertiary branches of each disk spine. The radial shields are long, concealed by disk plates and thin skin. The distal tips of the radial shields are exposed. The interradii are considerably swollen, ventrally covered with numerous similar spines (**Fig 1B)**. Areas adjacent to the genital slits are devoid of spines. Genital slits are long and narrow, gradually widened proximally without forming a distinct pouch. Each jaw bears a single wide apical papilla and four oral papillae, similar in shape and size, on both sides. The fifth thorny “oral papilla” which distinguishes the new species is the adoral shield papilla (or spine) (**Fig 1B and 1F)**, a juvenile feature which persists into adulthood in this species. The adoral papilla is often orientated perpendicular to the disk’s plane **(Fig 1F; Fig 2B)**, and thus different in appearance from the oral papillae. The teeth are massive, elongated and placed vertically one by one. The oral shield is wider than long, lozenge-shaped, the distal lobe is not evident **(Fig 1B)**. The lateral edges of the oral shield are distinctly attenuated. The adoral shield is wing-shaped laterally, widely adjoining the arm, slightly narrowing towards the mid-line of the jaws. The arm length is up to two and a half times the disk diameter. The dorsal arm plates are wider than long, sharply triangular proximally and convex distally, widely separated (up to the entire length of the dorsal arm plate) throughout the arm **(Fig 1C)**. Moniliform arms have conspicuous nodes; the lateral arm plates have a high lateral ridge, on which the large spine-articulations are placed. There is no spine-like protuberance on the lateral surface of the lateral arm plate **(Fig 3O and 3P)**. There are three spines on the first segment under the disk, six spines on the second segment, nine spines on the third (free) segment, 6–8 spines on the following free segments, six on the middle, 4–5 on the earlier distal segments and three spines on furthest distal segments. The dorsalmost spines on the proximal segments are the longest **(Figs 1C, 1E and 3D)**; the spines adjacent to the disk may reach three arm segments in length. Ventral spines are considerably shorter (**Fig 3I**). Dorsal spines are hollow through their entire length **(Fig 3G and 3H)** and bear thorns of varying number and length. The ventral spines have small cavities inside **(Fig 3J)**, are thornier and rather club-shaped. The ventral arm plates **(Fig 3C and 3T)** are wider than long, triangular proximally and convex distally, widely separated (up to the entire length of the ventral arm plate) throughout the arm **(Fig 1D)**. The tentacle pores are small and bear a single tentacle scale. The proximal segments bear markedly thorny tentacle scales, similar in shape to the ventral arm spines and adoral shield papillae. Further segments possess less thorny tentacle scales. The tentacle scales are shorter than the arm spines but conspicuous and capable of covering the whole tentacle pore.

Variability of adult characters: The 2,794 paratypes possess the essential external diagnostic characters of the new species: a massive finely thorny, club-shaped papilla, placed on each adoral shield, and acutely attenuated lateral sides of the oral shield. A large sample of 901 paratypes (NSMT E–7638) has been thoroughly investigated to detect variation of different external characters. The following variations were found: 84 specimens display 5–6 asymmetrically placed oral papillae on one side of some half-jaws instead of strictly 4 symmetrically placed oral papillae on both half-jaws; 10 specimens display 5–6 close to symmetrically placed oral papillae on both half-jaws; one specimen has 9 oral papillae and one specimen with only 3 oral papillae on one side of some of the half jaws. Three specimens have only four arms and two specimens with six arms were found. All display the diagnostic characters of *O*. *kokusai*, however the six armed specimens have more tightly “packed” oral frames and more narrow oral shields because of the additional interradius. About 20 specimens have an unusual-shaped oral shield, in some cases with the width almost similar to height, but they always have the characteristic attenuated lateral sides.

Internal and microstructural adult characters (Figs 2–6): Each disk spine has a long pedicel bearing an elaborate rosette of long forked thorns **(Fig 2A)**. The radial shield is an elongated plate, with a slightly elevated articulation surface **(Fig 4A and 4E)**. The articulation surface of the genital plate has a slightly elevated condyle. The abradial genital plate is well defined. Jaws are relatively high and slightly elongated. Adradial sides of the jaws bear folds distally. The adoral papilla bears a few strong and numerous fine spines **(Figs 2B, 5C and 5D)** and is placed at the upper corner of each adoral shield **(Fig 6D–6F)**. The dental plate has a vertical series of elongated sockets **(Fig 5E and 5F)**. Arm spine articulations on the holotype (NSMT E–3188) are of the ophiacanthid type [31, 57, 58] with a distinct sigmoidal fold **(Fig 3A, 3E and 3O–3Q)**. Vertebrae are rather long, with reduced zygospondylous articulation in proximal and some middle segments **(Figs 3M and 5K)**, becoming streptospondylous toward distal segments **(Fig 3S)**. The dorso-distal vertebral keel (distal projection of the dorsal side of vertebrae) is widened and distinctly divided into two lobes **(Figs 3K, 3M and 5I)**. Vertebral dorsal median groove and lateral curved grooves [31, 59] are very distinct **(Fig 3K)**. Podial basins are small.

#### Temperature preferences

*O*. *kokusai* has been predominantly collected from sites with mean bottom temperature below 10°C (Fig 7B, 7C and 7E). There are no records from localities with mean annual bottom temperature values over 15°C (Fig 7D and 7F) or below 5°C (Fig 7A). The mean maximal value is 14.8°C and mean minimal value is 6.4°C (Fig 8C and 8D).

Bathymetric range: The species has been found at depths of 151–618 m. It most commonly occurs between 300 m and 500 m. At its uppermost bathymetric limit (150–250 m) it overlaps with *O*. *rhachophora*, and at the lowermost limit (500–600 m) it co-exists with *O*. *trachybactra* **(Fig 8A)**. The bathymetric and thermal differences were confirmed with high statistical support (Fig 8B and 8D) under nonparametric Mann-Whitney rank sum test using SigmaPlot for Windows (Version 11.0) software (see below for details).

#### Geographic range

The southernmost limits of this species are Yakushima Id. and Tanegashima Id., Kagoshima Pref. (30° 4,6' N 130° 55,7' E), and the northernmost limit is SE of Taitosaki, Bōsō Peninsula, (35° 09,60' N 140° 49,40' E). It most commonly occurs along the Pacific side of central Honshū, especially in Suruga Bay and Sagami Bay (Fig 7E). It has not been found off Northern Honshū, Hokkaido (Fig 7A), or in the Sea of Japan.

Remarks: Distinguishing features of the new species see under Discussion.

**Fig 9:**
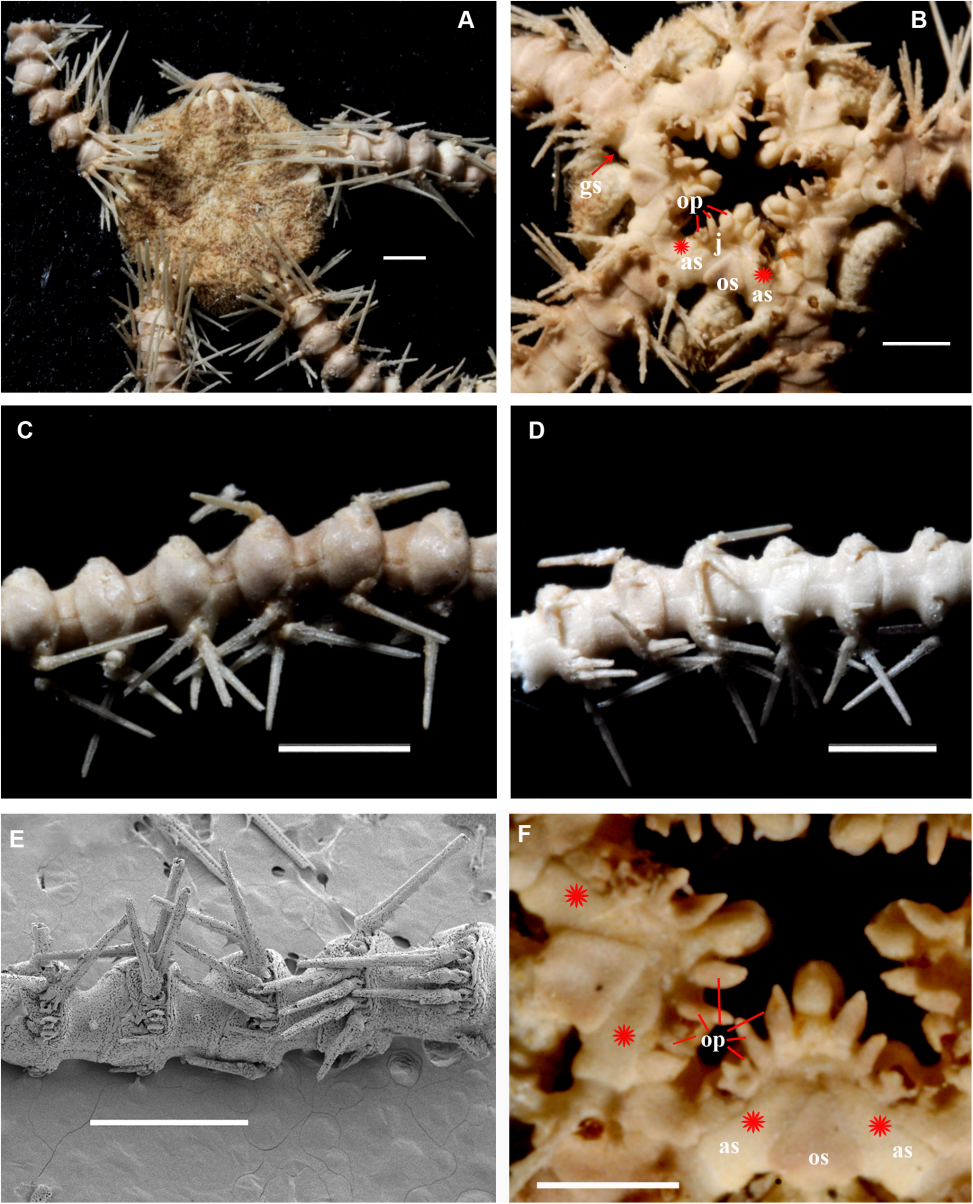
*Ophiacantha rhachophora* H.L. Clark, 1911, holotype USNM 25630 from Gotō Islands (ca. 100 km W to Nagasaki Prefecture), 5.6 mm dd, external views. **A,** dorsal view; **B,** ventral view; **C,** proximal arm segments, dorsal view; **D,** proximal arm segments, ventral view; **E,** proximal arm segments, lateral view, SEM; **F,** oral frame, details. as, adoral shields; gs, genital slit; j, jaws; op, oral papillae; os, oral shield; red asterisks indicate the former position of the adoral shield papillae during early ontogeny (i.e. absence of the distalmost oral papillae at the adoral shields in adult state). Scales bars, 1 mm (A–F).

**Fig 10:**
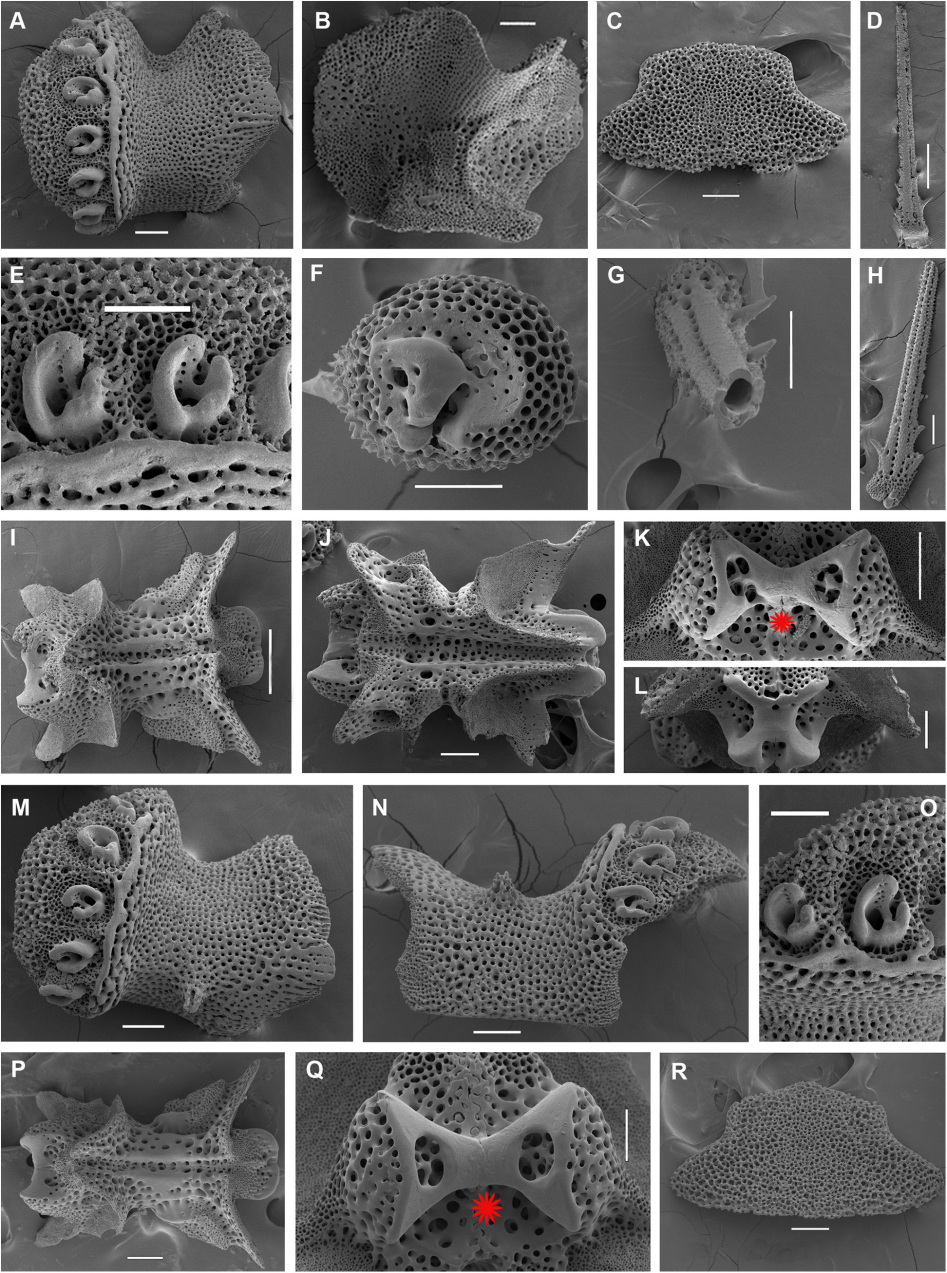
*Ophiacantha rhachophora* H.L. Clark, 1911, holotype USNM 25630, 5.6 mm dd, details, SEM. **A–L, proximal segments. A,** lateral arm plate; **B,** same, inside view; **C,** ventral arm plate; **D,** dorsal spine; **E,** arm spine articulations; **F,** spine, ventral view; **G,** hollow dorsal spine, transversally sectioned at middle part; **H,** ventral spine; **I,** vertebra, dorsal view; **J,** same, ventral view; **K,** same, distal view; **L,** same, proximal view; **M–R, middle segments. M–N,** lateral arm plates showing spine-like protuberance; **O,** arm spine articulations; **P,** vertebra, dorsal view; **Q,** same, distal view; **R,** ventral arm plate. Red asterisks indicate the absence of the vertebral condyle (streptospondylous articulation) on both proximal and distal vertebrae. Scales bars, 0.05 mm (E, F, Q), 0.1 mm (A–C, G, H, J, K–R), 0.2 mm (D, I).

**Fig 11:**
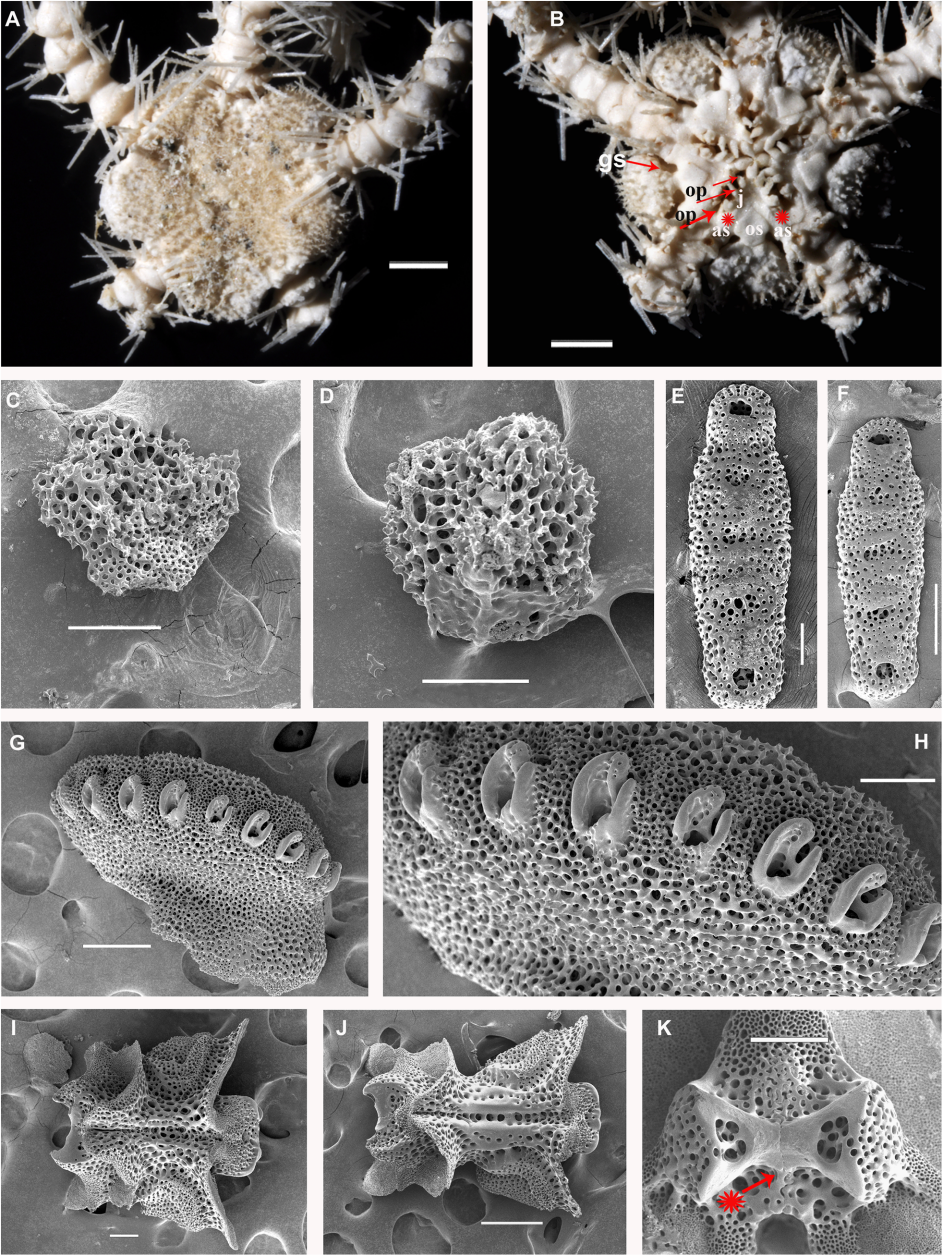
*Ophiacantha rhachophora* H.L. Clark, 1911, NSMT E–7614 from Sagami Bay, 4.5 mm dd, external views and details, SEM. **A,** dorsal view; **B,** ventral view; **C,D,** lobed, not thorny, distalmost oral papilla placed on the jaw instead of adoral shield; **E, F**, dental plates; **G,** third lateral arm plate (first free proximal segment) closely adjacent to the disk showing no striated elevation near articulations; **H,** arm spine articulations; **I,** proximal vertebra, dorsal view; **J,** middle vertebra, dorsal view; **K,** proximal vertebra, distal view. as, adoral shields; op, oral papillae; gs, genital slit; j, jaws; op, oral papillae; os, oral shield; red asterisk indicates the absence of the vertebral condyle (streptospondylous articulation) on proximal vertebrae. Scales bars, 0.1 mm (C, D, G–K); 0.2 mm (E, F), 1 mm (A, B).

*Ophiacantha rhachophora* H.L. Clark, 1911. (Figs 2 and 7–11)

*Ophiacantha rhachophora–*H.L. Clark, 1911: 201–202, fig. 92 [56], H.L. Clark, 1913: 217 [60], H.L. Clark, 1915: 204 [61] (partim., including *O*. *kokusai* and *O*. *trachybactra*).–Matsumoto, 1917: 119–120, fig. 30 [62] (partim., including *O*. *kokusai*.)–Murakami, 1942: 5–6 [63] (partim., including *O*. *kokusai*).–Djakonov, 1954: 32–33, fig.10 [64] (partim., including *O*. *kokusai* and *O*. *trachybactra*).–A.M. Clark, 1965: 41 [65].–Kyte, 1977: 55–57, fig. 1–2 [66] (partim., including *O*. *kokusai*).–Irimura, 1991: 126, fig. 49 [67].–Fujita, Ishida & Irimura, 1997: 258–259 [34].–Fujita & Irimura, 2005: 366 [35] (partim., including *O*. *kokusai*).

#### Holotype

USNM 25630 (dry) **(Figs 9 and 10)**, R/V “Albatross”, sta. 4902, 10-08-1906, Gotō Islands, 32° 30' 50" N 128° 34' 40" E., 254 m, gray sand, broken shells, bottom temperature 11.6°C.

#### Paratypes

1 dry paratype MCZ 3223, R/V “Albatross”, sta. 4902, 10.08.1906, Gotō Islands, 32° 30' 50" N 128° 34' 40" E., 254 m, gray sand, broken shells, bottom temperature 11.6°C. 1 dry paratype USNM 25987, R/V “Albatross”, sta. 4903, 10.08.1906, Gotō Islands, lat. 32° 31' 10" N 128° 33' 20" E, 195–254 m, gray sand, broken shells, bottom temperature 11.6°C.

#### Other material

(See S1 Appendix)

#### Description of the adult holotype

The disk is 5.6 mm in diameter, not indented interradially. The disk scales are concealed by thin skin and numerous spines (**Fig 9A**). The long radial shields do not form elevated bars, their distal tips are exposed. The interradii are considerably swollen, ventrally covered with numerous similar spines **(Fig 9B).** Areas adjacent to the genital slits are devoid of spines. Genital slits are wide, rapidly widening proximally and forming a distinct pouch. Each jaw bears one wide apical papilla and three oral papillae on both sides. Two more proximal oral papillae are conical and smooth, whereas the third distalmost oral papillae bear 3–4 massive blunt tooth-like lobes **(Fig 9B and 9F)**. The fourth adoral shield “oral papilla” is completely absent. The teeth are massive, elongated and placed vertically in a single column. The oral shield is longer than wide, distinctly spade-shaped with a small distal lobe (**Fig 9F)**. The adoral shield is wing-shaped laterally, widely adjoining the arm, slightly narrowing towards the mid-line of the jaws. The arm length is up to two and half times the disk diameter. The dorsal arm plates are wider than long, sharply triangular proximally and convex distally, widely separated (up to the entire length of the dorsal arm plate) throughout the arm **(Fig 9C)**. Arms have conspicuous nodes and lateral arm plates have a high lateral ridge, on which the large spine articulations are placed. There is a spine-like protuberance on the lateral surface of the lateral arm plate (**Fig 10M and 10N**). There are three spines on the first segment under the disk, 6–7 spines on the following free segments, 5–6 on the middle, 4–5 on the earlier distal segments. The dorsalmost spines on the proximal segments are the longest **(Figs 9A, 9E and 10D)**. The spines adjacent to the disk may reach the length of three arm segments. The ventral spines are considerably shorter. Dorsal spines are hollow and bear thorns of varying number and length (**Fig 10D, 10G and 10H**). The ventral spines are thornier. The ventral arm plates are wider than long, triangular proximally and convex distally, widely separated (up to the entire length of the dorsal arm plate) throughout the arm **(Fig 9D)**. The tentacle pores are small and bear a single tentacle scale. Proximal scales are thorny, becoming smoother distally. The tentacle scales are shorter than the ventral arm spines but conspicuous and capable of covering the whole tentacle pore.

Variability: Two hundred and twenty specimens, including the type series, were studied. Most are essentially similar to the holotype **(Figs 2D and 11)**. The oral shield is usually distinctly longer than wide, but in some specimens it may become wider, though less so than the wide oral shield of *O*. *kokusai* sp. nov. Rarely in few specimens additional oral papillae are present. Among 118 studied specimens of *O*. *rhachophora* from one particularly large lot (NSMT E–1540), only 8–10 specimens had additional papillae, up to 5 per half-jaw; mostly occurring on just one half-jaw, thus making the number of papillae per jaw asymmetric. No specimens of *O*. *rhachophora* were found with spine-like papillae placed fully on the adoral shields. The shape of the distalmost oral papilla can vary but usually bears a few teeth-like cusps. It may also be smooth. Cuspidate and smooth distalmost papillae may co-occur in the same interradius.

Internal and microstructural adult characters (Figs 2C, 2D, 6A–6C, 10 and 11): Each disk spine with a long pedicel that bears an elaborate rosette of long forked thorns **(Fig 2C)**. The radial shield is an elongated plate, with a slightly elevated articulation surface. The articulation surface of the genital plate has a slightly elevated condyle. The abradial genital plate is well defined. Jaws are relatively high and slightly elongated. Adradial sides of the jaws bear folds distally. The adoral shield papilla is absent **(Figs 2D and 6A–6C)**. The dental plate has a series of elongated sockets **(Fig 11E and 11F)**. Arm segments of the holotype of *O*. *rhachophora* (USNM 25630) were investigated using SEM **(Figs 9E and 10)**. Arm spine articulations are of the ophiacanthid type [31, 57, 58] with a distinct sigmoidal fold **(Fig 10A, 10E and 10M–10O)**. Vertebrae are rather long, with traces of zygospondylous articulation on the most proximal segments only (**Fig 10K**); most of the vertebrae along the arm are completely streptospondylous **(Figs 10Q and 7K)**. The dorsal vertebral keel distally is narrowed to form an arrow-shaped structure **(Figs 10I, 10P, 11I and 11J)**, which is insignificantly lobed. Vertebral dorsal median groove and lateral curved grooves [31, 59] are distinct **(Figs 10I and 11I)**. Podial basins are small.

Temperature preferences: *Ophiacantha rhachophora* rarely occurs at localities with mean annual seafloor temperature below 10°C. Mean maximal temperature is 20.63°C. Mean minimal value is 9.5°C (Fig 8C and 8D).

Bathymetric range: The species has been found at depths of 50–487 m, most commonly between 150 m and 250 m. Only four juvenile specimens were found at one site below 300 m in the southernmost part of the range (Okinawa Prefecture). This site had abnormally high temperatures (17°C) in the month (May) the specimens were collected (Fig 7D and 7F). The multiyear collection data agree with bottom temperature measurements since *O*. *rhachophora* in the northern part of its range was never found deeper than 300 m **(Figs 7 and 8)**. *Ophiacantha kokusai* sp. nov. which instead typically inhabits depths below 300 m off central Honshū (Fig 8) was not found to co-occur with *O*. *rhachophora* at similar depths in the southernmost limits of the distribution of the latter species. In addition, *O*. *rhachophora* occurs in considerable quantities at shallow depths (to 50 m) (Fig 8A), whereas *O*. *kokusai* was never found shallower than 150 m.

Geographic range: The southernmost limit of this species is Aguni East, Okinawa Pref. **(**26° 30,31' N 127° 26,13' E) (Fig 7D and 7F) and the northernmost limit is off Chikura, Bōsō Peninsula, Chiba Pref., (34° 55,1' N 140° 2,7' E) (Fig 7E). It was not found off Northern Honshū, Hokkaido (Fig 7A) or in the Sea of Japan. The range of *O*. *rhachophora* thus extends to Okinawa, significantly more southern than *O*. *kokusai*. Due to numerous misidentifications of *O*. *rhachophora* (see discussion below) records considerably exceeding the known range (e.g. H.L.Clark’s [56] from California) may refer to other species.

*Ophiacantha trachybactra* H.L. Clark, 1911. (Figs 2, 7, 8 and 12–19)

**Fig 12:**
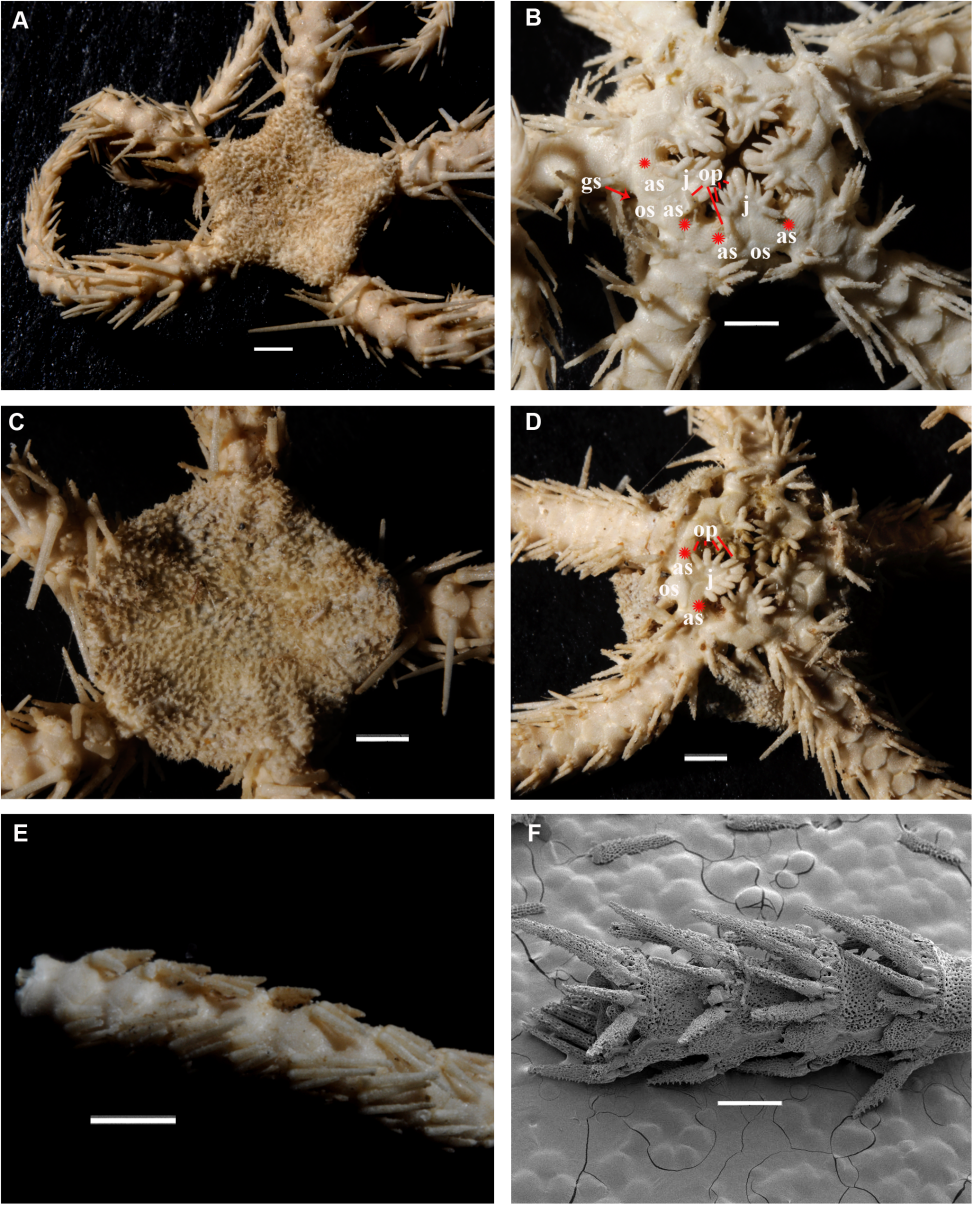
*Ophiacantha clypeata* Kyte, 1977 (new synonym of *O*. *trachybactra* H.L. Clark, 1911), type material, external views. **A,** holotype USNM 26241 from Bowers Bank, Bering Sea, 5.2 mm dd, dorsal view; **B,** same, ventral view; **C,** paratype USNM 26698 from Bowers Bank, Bering Sea, 6.5 mm dd, dorsal view; **D,** same, ventral view; **E,** proximal arm segments, lateral view, SEM; **F,** proximal arm segments, lateral view, details, SEM. ap, adoral shield papillae; as, adoral shields; gs, genital slit; j, jaws; op, oral papillae; os, oral shield; red asterisks indicate the absence of the adoral shield papillae. Scales bars, 0.5 mm (F), 1 mm (A–E).

**Fig 13:**
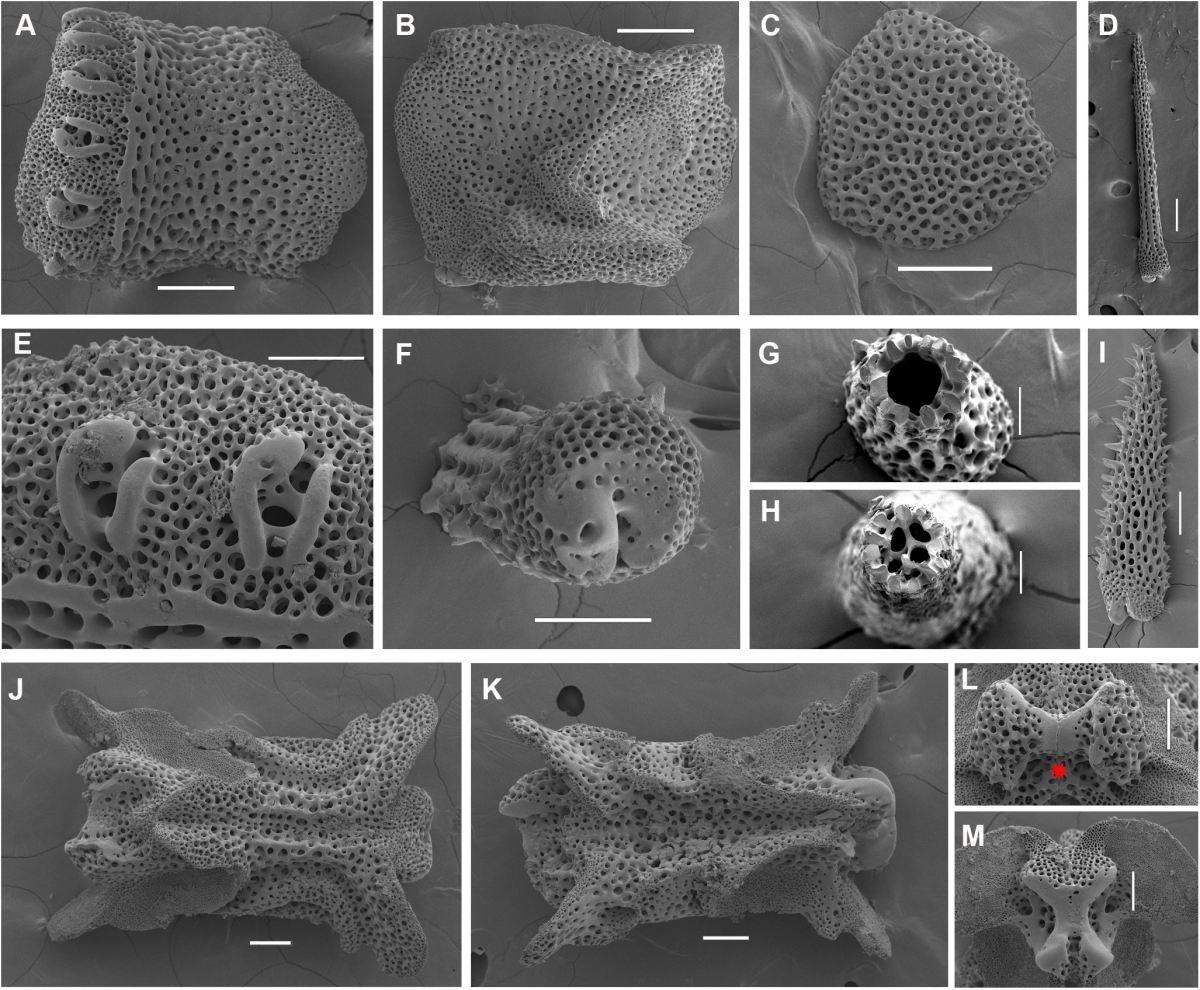
*Ophiacantha clypeata* Kyte, 1977 (new synonym of *O*. *trachybactra* H.L. Clark, 1911), details of the paratype USNM 26698, middle segments, SEM. **A,** lateral arm plate; **B,** same, inside view; **C,** ventral arm plate; **D,** dorsal spine; **E,** arm spine articulations; **F,** spine, ventral view; **G,** dorsal spine, transversely sectioned at the base showing a single large cavity; **H,** dorsal spine, transversely sectioned in the middle showing several small cavities**; I,** ventral spine; **J,** vertebra, dorsal view; **K,** same, ventral view; **L,** same, distal view; **M,** same, proximal view. Red asterisk indicates the absence of vertebral condyle (streptospondylous articulation) throughout the arms. Scales bars, 0.05 mm (G), 0.1 mm (E, F, I–M), 0.2 mm (A–D).

**Fig 14:**
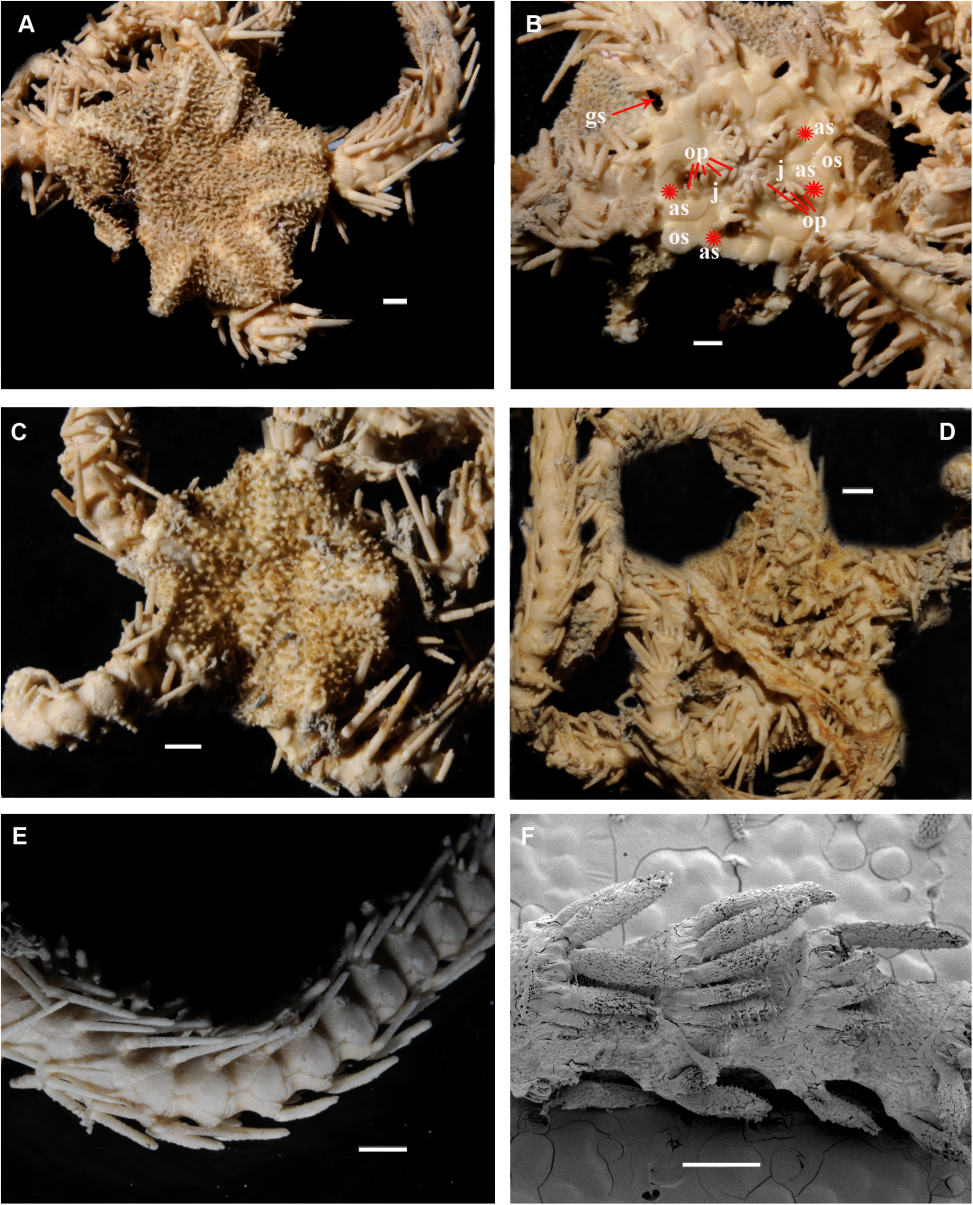
*Ophiacantha trachybactra* H.L. Clark, 1911, type material, external views. **A,** holotype USNM 25649, 12.7 mm dd, off Cape Terpeniya, Sakhalin Id., Okhotsk Sea, dorsal view; **B,** same, ventral view; **C,** paratype USNM 25694, Shumagin Bank, Alaska, 7.2 mm dd, dorsal view; **D,** same, ventral view; **E,** same, proximal arm segments, dorsal view; **F,** same, latero-ventral view, SEM. as, adoral shields; gs, genital slit; j, jaws; op, oral papillae; os, oral shield; red asterisks indicate the absence of the adoral shield papillae. Scales bars, 0.5 mm (F), 1 mm (A–E).

**Fig 15:**
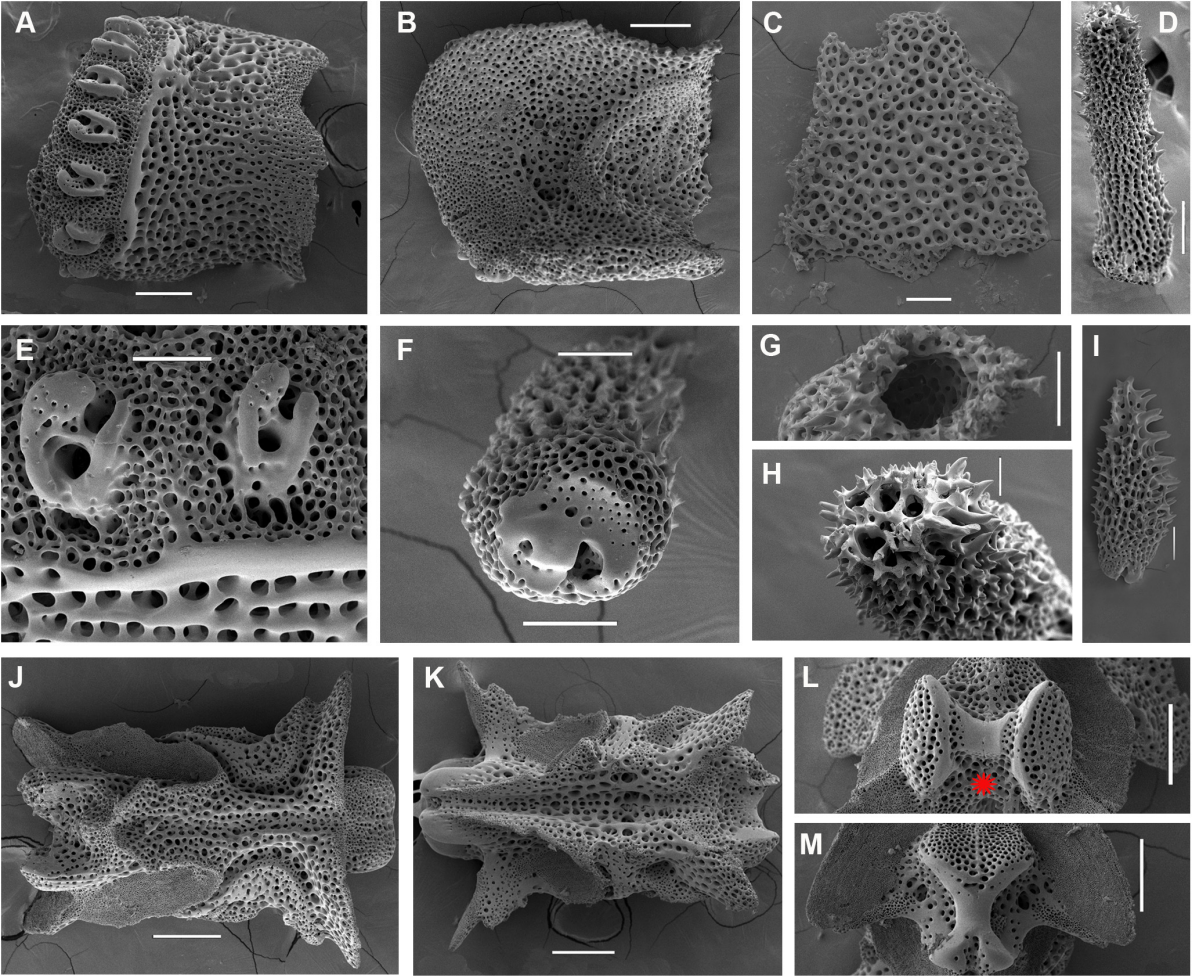
*Ophiacantha trachybactra* H.L. Clark, 1911, details of the paratype USNM 25694, details, middle segments, SEM. **A,** lateral arm plate; **B,** same, inside view; **C,** ventral arm plate; **D,** dorsal spine; **E,** arm spine articulations; **F,** spine, ventral view; **G,** dorsal spine, transversely sectioned at the base showing a single large cavity; **H,** dorsal spine, transversely sectioned in the middle showing several small cavities**; I,** ventral spine; **J,** vertebra, dorsal view; **K,** same, ventral view; **L,** same, distal view; **M,** same, proximal view. Red asterisk indicates the absence of the vertebral condyle (streptospondylous articulation) throughout the arms. Scales bars, 0.05 mm (H), 0.1 mm (C, E, F, G, I), 0.2 mm (A, B, D, J–M).

**Fig 16:**
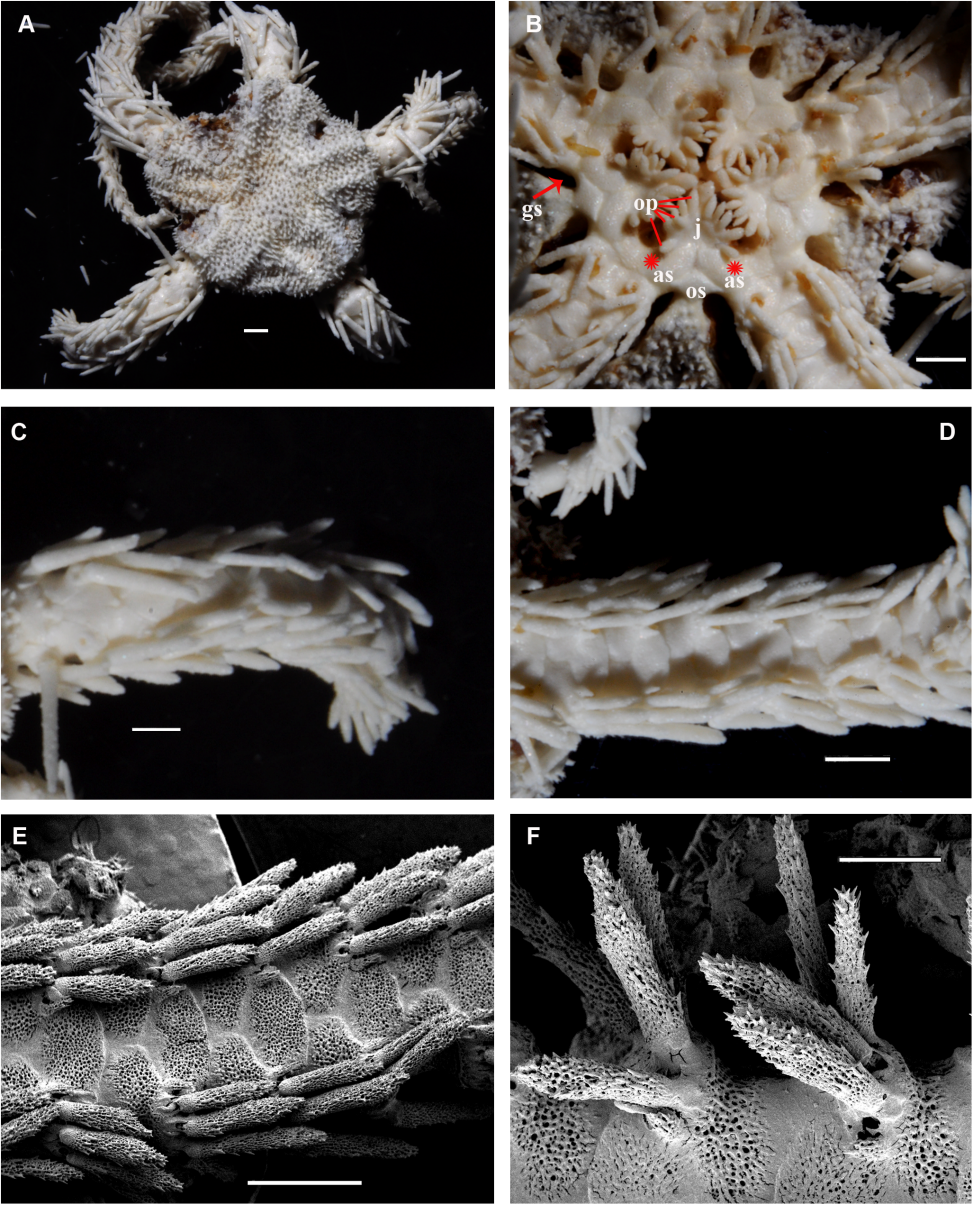
*Ophiacantha trachybactra* H.L. Clark, 1911, adult specimen 10.9 mm disk diameter NSMT E–7557, sta. KT-93-15 (M 3), off Sanriku, northern Honshū, external views. **A,** dorsal view; **B,** ventral view; **C,** proximal arm segments, dorsal view; **D,** proximal arm segments, ventral view; **E,** proximal arm segments, ventral view, SEM; **F,** proximal arm segments, ventral view, details, SEM. as, adoral shields; gs, genital slit; j, jaws; op, oral papillae; os, oral shield; red asterisks indicate the absence of the adoral shield papillae. Scales bars, 0.5 mm (F), 1 mm (A–E).

**Fig 17:**
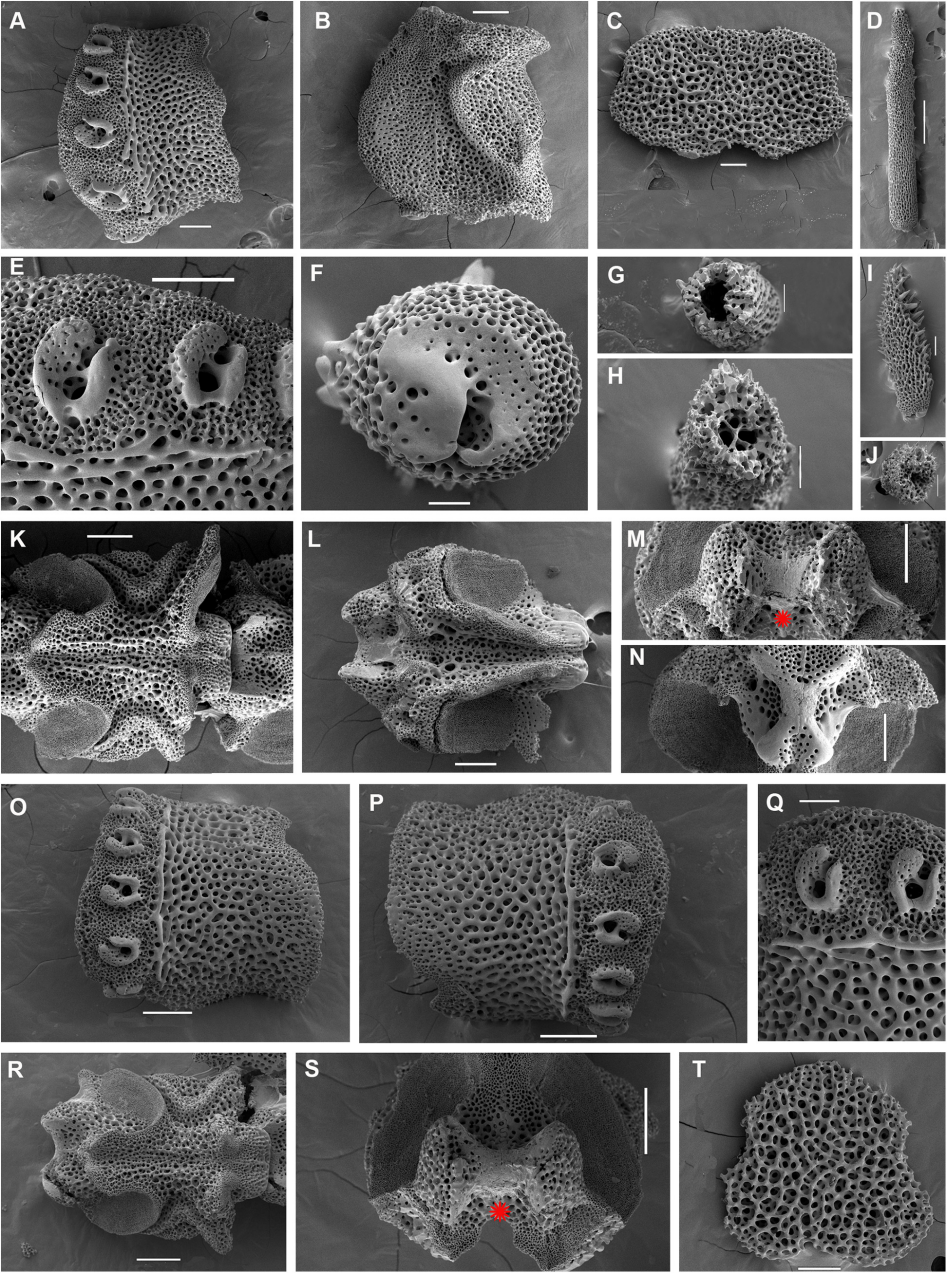
*Ophiacantha trachybactra* H.L. Clark, 1911, adult specimen 10.9 mm disk diameter NSMT E–7557, sta. KT-93-15 (M 3), off Sanriku, northern Honshū, details, SEM. **A–N, proximal segments. A,** lateral arm plate; **B,** same, inside view; **C,** ventral arm plate; **D,** dorsal spine; **E,** arm spine articulations; **F,** spine, ventral view; **G,** dorsal spine, transversely sectioned at the base showing a single large cavity; **H,** dorsal spine, transversely sectioned in the middle showing several small cavities; **I,** ventral spine; **J,** ventral spine sectioned in the middle showing small cavities; **K,** vertebra, dorsal view; **L,** same, ventral view; **M,** same, distal view; **N,** same, proximal view; **O–T, middle segments. O–P,** lateral arm plates showing no additional protuberance; **Q,** arm spine articulations; **R,** vertebra, dorsal view; **S,** same, distal view; **T,** ventral arm plate. Red asterisks indicate the absence of the vertebral condyle (streptospondylous articulation) throughout arms. Scales bars, 0.05 mm (F), 0.1 mm (C, E, G, H, I, J, Q, T), 0.2 mm (A, B, K, L, M, N, O, P, R, S), 0.5 mm (D).

**Fig 18:**
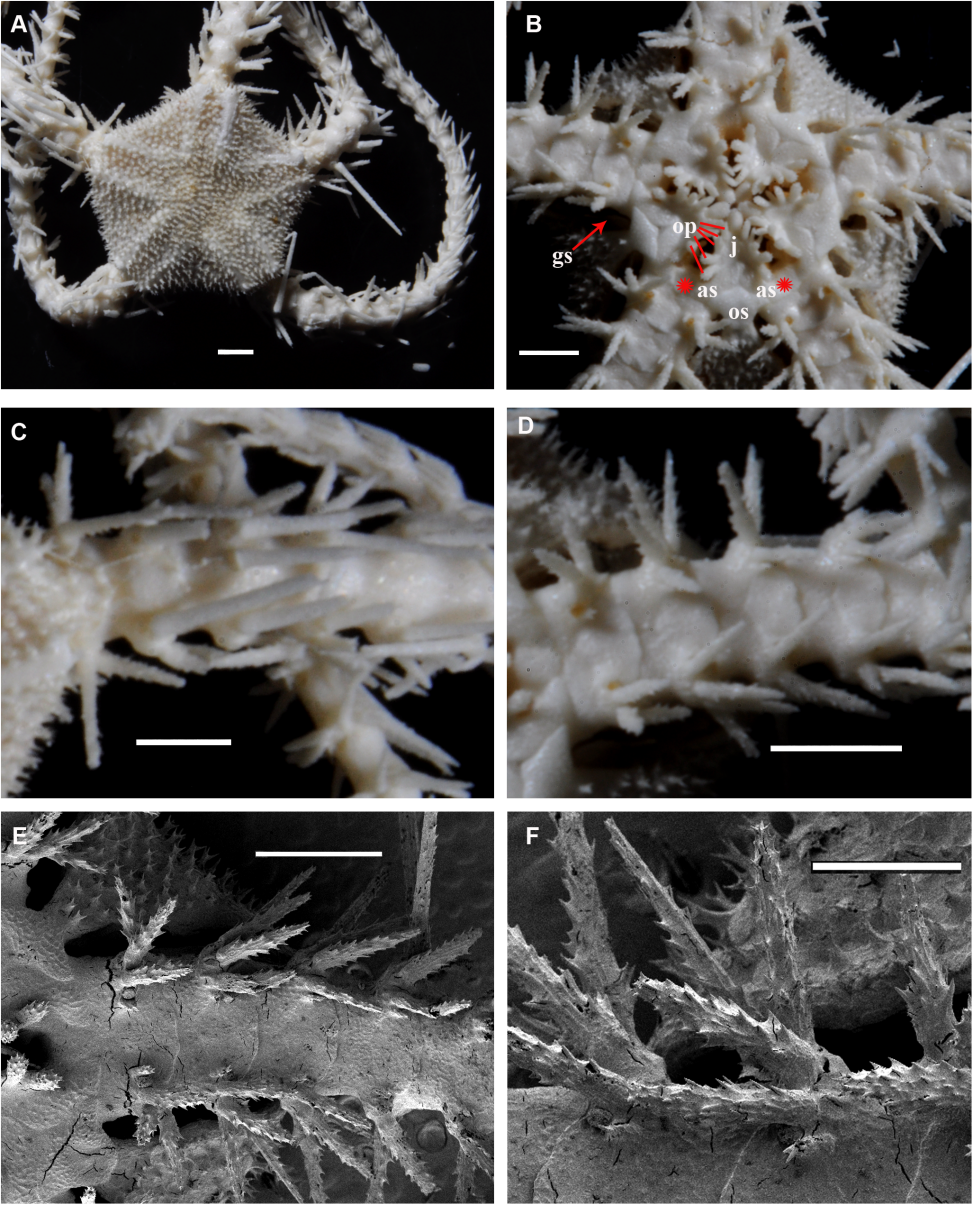
*Ophiacantha trachybactra* H.L. Clark, 1911, subadult specimen 6.8 mm disk diameter NSMT E–7557, sta. KT-93-15 (M 3), off Sanriku, northern Honshū, external views. **A,** dorsal view; **B,** ventral view; **C,** proximal arm segments, dorsal view; **D,** proximal arm segments, ventral view; **E,** proximal arm segments, ventral view, SEM; **F,** proximal arm segments, ventral view, details, SEM. as, adoral shields; gs, genital slit; j, jaws; op, oral papillae; os, oral shield; red asterisks indicate the absence of the adoral shield papillae. Scales bars, 0.5 mm (F), 1 mm (A–E).

**Fig 19:**
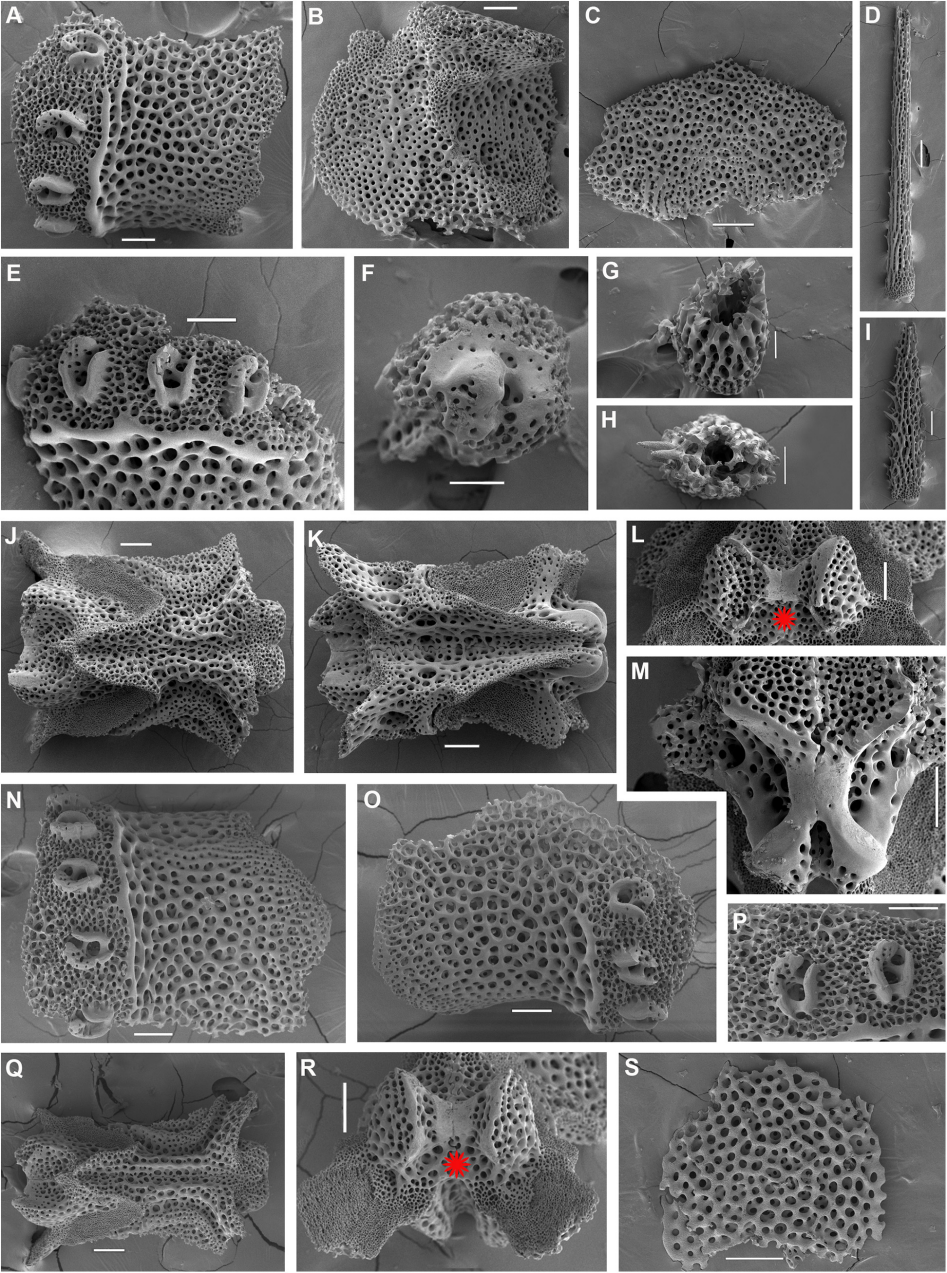
*Ophiacantha trachybactra* H.L. Clark, 1911, subadult specimen 6.8 mm disk diameter NSMT E–7557, sta. KT-93-15 (M 3), off Sanriku, northern Honshū, details, SEM. **A–M, proximal segments. A,** lateral arm plate; **B,** same, inside view; **C,** ventral arm plate; **D,** dorsal spine; **E,** arm spine articulations; **F,** spine, ventral view; **G,** dorsal spine, transversely sectioned at the base showing a single large cavity; **H,** dorsal spine, transversely sectioned in the middle showing several small cavities; **I,** ventral spine; **J,** vertebra, dorsal view; **K,** same, ventral view; **L,** same, distal view; **M,** same, proximal view; **N–T, middle segments. N,O,** lateral arm plates showing no additional protuberance; **P,** arm spine articulations; **Q,** vertebra, dorsal view; **R,** same, distal view; **S,** ventral arm plate. Red asterisks indicate the absence of the vertebral condyle (streptospondylous articulation) throughout the arms. Scales bars, 0.05 mm (F, G, H), 0.1 mm (A, B, C, E, I–S), 0.2 mm (D).

*Ophiacantha trachybactra* H.L. Clark, 1911a: 206–207, fig. 96 [56].–Djakonov, 1954: 30–31 [64].–Belyaev & Litvinova, 1976: 137 [68].–Irimura, 1991: 128, fig. 51 [67].

*Ophiolebes trachybactrus* H.L. Clark, 1915: 195 [66].

*Ophiacantha clypeata* Kyte, 1977: 57–59, fig. 3–4 [61], **syn. nov.**

#### Holotype

USNM 25649 (dry), R/V “Albatross”, sta. 5029, 28-09-1906, Okhotsk Sea, Sakhalin Id., 48° 22' 30" N 145° 43' 30" E., 805 m, black sand, gravel, bottom temperature 1.8°C.

#### Paratypes

1 dry paratype USNM 26816, R/V “Albatross”, sta. 4781, 07.06.1906, Bering Sea, 52° 14' 30" N 174° 13' E., 882 m, fine gray sand, pebbles, bottom temperature 3.7°C. 1 dry paratype USNM 25694, R/V “Albatross”, sta. 3338, 28.08.1890, Alaska Peninsula, Shumagin Islands, Shumagin Bank, 54° 19' N 159° 40' W, 1143 m, green mud, sand, bottom temperature 2.9°C. 2 dry paratypes MSZ 3225, R/V “Albatross”, sta. 3338, 28.08.1890, Alaska Peninsula, Shumagin Islands, Shumagin Bank, 54° 19' N 159° 40' W, 1143 m, green mud, sand, bottom temperature 2.9°C.

#### Type material of *O*. *clypeata* Kyte, 1977

Holotype (dry) USNM 26241 **(Fig 12A and 12B)**, R/V “Albatross”, sta. 4772, 04.06.1906, Bering Sea, Aleutian Ids, Bowers Bank, 54° 30' 30" N 179°14' E., 629 to 681 m, green brown sand. 2 dry paratypes USNM 26698 **(Figs 12C–12F and 13)**, station 4771, 04.06.1906, Bering Sea, Aleutian Ids, Bowers Bank, 54° 30’N 179° 17' E., 779 m, broken shells, bottom temperature 38 1. 11 ethanol paratypes USNM 27016, station 4775, 04.06.1906, Bering Sea, Aleutian Ids, Bowers Bank, 54° 33' 30" N 178 44' E, 1068 m, green mud, black specks, foraminifera, bottom temperature 37.2. 2 dry paratypes MSZ 3224, station 4775, 04.06.1906, Bering Sea, Aleutian Ids, Bowers Bank, 54° 33' 30" N 178 44' E., 1068 m, green mud, black specks, foraminifera, bottom temperature 37.2.

#### Other material

(See S1 Appendix)

#### Description of the adult holotype of *Ophiacantha trachybactra* (USNM 25649)

The disk is 12.7 mm in diameter, not indented interradially. The disk scales are concealed by thick skin and numerous short spines **(Fig 14A)**.The long radial shields form elevated bars, their distal tips are concealed by skin **(Fig 14A)**. The interradii are swollen, ventrally covered with numerous similar spines. Areas adjacent to the genital slits are covered with spines. The genital slits are narrow, gradually widened proximally and form an elongate opening **(Fig 14B)**. Each half-jaw bears 5–7 oral papillae. The apical papilla is poorly preserved in the holotype. Oral papillae are similar in size and shape, conical and have a rough surface. The adoral shield papilla is completely absent **(Fig 14B)**. The teeth are massive, elongated and placed vertically in one column. The oral shield is lozenge-shaped, similar in length and width, a distal lobe is not evident **(Fig 14B)**. The adoral shield is wing-shaped laterally, widely adjoining the arm, slightly narrowing towards the mid-line of the jaws. The arm length is up to three times the disk diameter. The dorsal arm plates are slightly wider than long, sharply triangular proximally and convex distally, widely separated (up to the entire length of the dorsal arm plate) throughout the arm **(Fig 14A)**. Arms do not form conspicuous nodes. The lateral arm plates have a lateral ridge, on which the large spine articulations are placed. There are three spines on the first segment under the disk, 4–5 spines on second and third segments under the disk, 6–7 spines on following proximal free segments, six on the middle, 4–5 on the earlier distal segments and 3 spines on the most distal segments. The dorsalmost spines on the proximal segments are the longest **(Fig 14A)**. The spines adjacent to the disk may reach 2.5 arm segments in length. The ventral spines are considerably shorter. Spines are only partially hollow (as in paratype on Fig 15G). Some dorsalmost spines are semi-solid inside with small holes (as in paratype on Fig 15H). Dorsalmost spines bear few fine spinelets. The ventral spines are thornier and distinctly club-shaped. Toward the middle and especially on distal segments, the ventralmost spines are transformed into comb-shaped structures, sometimes with a small apical hook. The ventral arm plates are wider than long, oval to quadrangular proximally, and triangular on distal segments. On both proximal and distal segments, the proximal side of the ventral arm plates is pointed and the distal side has a distinct pit medially. The ventral arm plates are slightly separated proximally and up to the length of the segment distally (Fig 14B). The tentacle pores are small and bear a single tentacle scale. The tentacle spine is small, conical, bears a few thorns and is similar along the arm. The scales are incapable of covering the whole tentacle pore.

Variability: About 130 specimens, including type series were studied. All of them share with the holotype (USNM 25649) such features as short, not branched, disk spines, relatively thick skin present both dorsally and ventrally on the disk and arms, club-shaped ventral arm spines (in adults), distal comb-shaped arm spines (both adults and subadults), and a small slightly thorny spiniform tentacle scale (Figs 12, 14 and 16). The oral shield is lozenge-shaped, wider than long, with only slightly attenuated lateral edges, and is similar in the majority of the studied specimens (Figs 12B, 12D and 16B). However, in some exemplars, including the holotype, it is rather similar in width and length (Fig 14B).

Internal and microstructural adult characters (Figs 13, 15, 17, 18 and 19): Each disk spine has a short pedicel that bears a reduced rosette of short simple thorns (up to 4) **(Fig 2E)**. The radial shield is an elongated plate, with a slightly elevated articulation surface. The articulation surface of the genital plate has a slightly elevated condyle. The abradial genital plate is well defined. Jaws are relatively high and slightly elongated. Adradial sides of the jaws bear folds distally. The dental plate has a series of elongated sockets. The adoral shield papilla is absent **(Fig 2F)**. Arm segments of the two paratypes of *O*. *trachybactra* **(Fig 14E and 14F)**, and the holotype of *O*. *clypeata* **(Fig 12E and 12F)** and several sub-adult (6.8 mm dd) **(Fig 18E and 18F)** and adult (10.9 mm dd) non-type specimens (NSMT E–7557) **(Fig 16E and 16F)** were studied using SEM. Arm spine articulations are of the ophiacanthid type [31, 57, 58] with a distinct sigmoidal fold **(Figs 13A, 13E, 15A, 15E, 17A, 17E, 17O–17Q, 19A, 19E and 19N–19P)**. Vertebrae are rather long, completely strepspondylous, without any traces of zygospondylous articulation even on the most proximal segments **(Figs 13L, 15L, 17M, 19L and 19R)**. The dorsal vertebral keel distally is narrowed to form an arrow-shaped structure, which is blunt or more sharpened **(Figs 13J, 15J, 17K, 19J and 19Q)**. Vertebral dorsal median groove and lateral curved grooves [31, 59] are very distinct **(Figs 13J and 15J)**. Podial basins are small. The dorsal arm spines are massive and semi-solid with several small cavities in the middle part of the spine **(Figs 13G, 13H, 15G, 15H, 14G, 14H, 17G and 17H)**, instead of being entirely hollow (typical of the family Ophiacanthidae).

Temperature preferences: ***O*. *trachybactra*** inhabits temperatures below 5°C (**Figs 7A, 8C and 8D**). It rarely occurred in localities with annual bottom temperature over 5°C (Fig 7B). There are no records for this species that have mean annual bottom temperature values over 10°C (**Figs 7D, 7F, 8C and 8D**). Mean maximal value is 6.84°C. Mean minimal value is 1.83°C.

Bathymetric range: The species has been found at depths of 531–1990 m, most commonly occurring between 1,100 m and 1,250 m (see **Fig 8A and 8B** and Discussion for details). The species clearly prefers the bathyal zone below 1,000 m, but has an uppermost bathymetric limit (500–600 m) that may overlap with *O*. *kokusai* (Fig 8A).

Geographic range: The southernmost limit of this species is Eastern Sagami Bay (35° 9,26' N 139° 30,99' E– 35° 9,22' N 139° 31,05' E) and Miyake-jima Id. (Fig 7E), the northernmost limit in Japanese waters is Hokkaido, S off Akkeshi (42° 28‘ N 145° 04‘E) (Fig 7A). Commonly found off Northern Honshū (Pacific side). The northernmost range of this species extends to the Bering Sea (Aleutian Ids.), Alaska (Shumagin Id.) and Canada (Queen Charlott Sound). The holotype comes from off Sakhalin Id. (Terpeniya Cape, Okhotsk Sea).

## Discussion

### Comparison of *Ophiacantha kokusai* sp. nov. to other species

*Ophiacantha kokusai* is most similar to *O*. *rhachophora* H.L.Clark, 1911 which also possesses spiny distalmost oral papillae. However *O*. *rhachophora* invariably lacks the characteristic thorny adoral shield papillae **(Figs 2D and 9F)**. Further distinguishing external features of *O*. *kokusai* include the predominantly wider than long oral shield, with attenuated narrow lateral sides (*O*. *rhachophora* has a heart-shaped oral shield with rounded lateral edges), and complete absence **(Fig 3O and 3P)** of spine-like protuberances on the lateral surface of the lateral arm plate (majority of the studied specimens of *O*. *rhachophora*, including the holotype, possess at least 1–2 spine-like protuberances per arm; commonly more **(Fig 10M and 10N)**). Among internal characters, the shape of the vertebrae reliably distinguishes *O*. *rhachophora* from *O*.*kokusai*, even for poorly preserved specimens (that have lost their adoral shield papillae) or those with aberrant oral shield shapes. The distal end of the dorsal keel of the vertebrae of *O*. *kokusai* is broadened and characteristically bi-lobed **(Figs 3K and 4I)**, whereas on *O*. *rhachophora* it is distinctly arrow-shaped with a narrow distal tip **(Figs 10I and 11J)**. The shape of the vertebrae differs between the two species, from proximal to distal segments, but most reliably on the fifth to eighth vertebrae because some proximal vertebrae of *O*. *rhachophora* may also have a somewhat widened, though still arrow-shaped, distal keel (**Fig 11I**). Other minor, but reliable, differences between *O*. *rhachophora* and *O*. *kokusai* are the shape of the arm spine articulations on the lateral arm plates, details of the striation patterns on the proximal lateral arm plates adjacent to the disk, and the shape of the dental plate.

The species *O*. *trachybactra* can be distinguished from both *O*. *kokusai* and *O*. *rhachophora* by the shape of the disk spines (short with few slightly-branched thorns), concealed (instead of uncovered) distal tips of the radial shields, the shape of the distalmost oral papillae (never thorny or cuspidate), complete absence of the adoral shield papillae, club-shaped ventral arm spines (in adult specimens), shape of the arm spine articulations and the distal dorsal keel of the vertebrae (see also below). However, all three species share similar sub-streptospondylous (*O*. *kokusai* sp. nov.) or completely streptospondylous (*O*. *rhachophora*, *O*. *trachybactra*) vertebral articulation. All three species are more similar to each other at the late juvenile stage than as adults (see below). Diagnostic features of all three species are summarized in Table 1.

**Table 1:** Diagnostic characters of the *Ophiacantha kokusai*, *O*. *rhachophora* and *O*. *trachybactra*.

Species	Disk spines	Radial shields	Adoral shield papillae	Oral shield	Oral papillae number	Vertebral dorsal keel shape	Vertebral articulaton	Dorsal arm spines
***Ophiacantha kokusai* sp.nov.**	Long, branched	Distally exposed	Present	Lozenge-shaped with attenuated lateral edges	8+	Broadened, bilobed	Proximally reduced zygospondylous, streptospondylous from middle segments	Entirely hollow
***Ophiacantha rhachophora* Clark H.L., 1911**	Long, branched	Distally exposed	Absent	Spades-shaped with rounded lateral edges	6+	Narrowed, arrow-shaped	Streptospondylous through entire arm	Entirely hollow
***Ophiacantha trachybactra* Clark H.L., 1911**	Short, not branched	Distally concealed	Absent	Lozenge-shaped with abrupt lateral edges	8–10+	Elongated, slightly bulged	Streptospondylous through entire arm	Hollow at base of arms, semi-solid from middle of arms

#### The heterogeneity of the type series of *Ophiacantha rhachophora*

Our study reveals considerable heterogeneity within the original type series of *O*. *rhachophora*, suggesting that it actually includes three species: *O*. *rhachophora*, *O*. *kokusai* and *O*. *trachybactra* (see below and Table 2). The sole figure accompanying the type description of *O*. *rhachophora* (fig. 92 in [56]) clearly represents a sub-adult specimen of *O*. *trachybactra*, according to the shape of disk spines and distalmost oral papillae. Similar specimens were separated from the type series of *O*. *rhachophora* as a new species *O*. *clypeata* [66], however the substantial similarity between “*O*. *clypeata*” and sub-adult specimens of *O*. *trachybactra* remained unnoticed.

**Table 2:** Heterogeneity of the original types series of *Ophiacantha rhachophora* Clark H.L., 1911.

*Ophiacantha rhachophora* type series	Status, number of specimens	R/V “Albatross” station	Locality	Depth (m)	Actual placement
USNM 25630	Holotype (dry)	4902	Gotō Islands, Japan	254	*O*. *rhachophora*
MCZ 3223	1 dry paratype	4902	Gotō Islands, Japan	254	*O*. *rhachophora*
USNM 25987	1 dry paratype	4903	Gotō Islands, Japan	254	*O*. *rhachophora*
USNM 26048	2 dry paratypes	4893	Gotō Islands, Japan	174–194	*O*. *rhachophora*
USNM 26049	2 dry paratypes	3717	Honshū Island, Suruga Bay, Ose-zaki	115–183	? *O*. *rhachophora*
USNM 26059	1 dry paratype	3698	Honshū Island, Sagami Bay, Manazuru-zaki	280	? *O*. *kokusai* sp.nov.
USNM 26241	1 dry paratype	4772	Bering Sea, Aleutian Islands, Bowers Bank	629–681	*O*. *trachybactra*
USNM 26243	6 dry paratypes	4965	Honshū Island, Kii Strait, S of Shirahama	349	? *O*. *kokusai* sp.nov.
USNM 26605	2 ethanol paratypes	4976	Honshū Island, S of Shiono Misaki	995–997	*O*. *adiaphora* according to Kyte, 1977: 55 [66]
USNM 26607	3 ethanol paratypes	5091	Honshū Island, Sagami Bay, S of Joga Island	360	*O*. *kokusai* sp.nov.
USNM 26698	2 dry paratypes	4771	Bering Sea, Aleutian Islands, Bowers Bank	779	*O*. *trachybactra*
USNM 26704	2 dry paratypes	5091	Honshū Island, Sagami Bay, S of Joga Island	360	*O*. *kokusai* sp.nov.
USNM 26781	1 dry paratype	3750	Honshū Island, Sagami Bay, Suno Saki	152–256	Unknown
USNM 26985	3 dry paratypes	4809	Hokkaido Island, Shirakami Point	165–379	*O*. *adiaphora* according to Kyte, 1977: 55 [66]
USNM 27016	11 ethanol paratypes	4775	Bering Sea, Aleutian Islands, Bowers Bank	1068	*O*. *trachybactra*
MSZ 3224	2 dry paratypes	4775	Bering Sea, Aleutian Islands, Bowers Bank	1068	*O*. *trachybactra*

The confusion surrounding these three very common North Pacific brittle star species persisted, both in the literature and in museum collections. As result, important characters have been overlooked and one of the most common Japanese species of the genus *Ophiacantha* was never described and considered to be *O*. *rhachophora* [56, 60–68].

#### Bathymetric and thermal differentiation of *O*. *rhachophora* and *O*. *kokusai*

These two species show bathymetric and temperature preferences (see also above and **Figs 7 and 8**). The species diagnosed here as *O*. *rhachophora* is distributed on the outer continental shelf (50–200 m) with temperature preferences of 10–20°C, whereas *O*. *kokusai* is predominantly distributed on the upper continental slope (300–500 m) with lower temperature preferences (generally below 10°C, not higher than 15°C). Bathymetric outliers tend to have atypical temperature profiles (**Fig 7**). For example, in the northern part of the range of *O*. *rhachophora*, off central Honshū, the mean annual temperatures above 10°C occur considerably shallower (100–200 m) than in the southern part of the range (compare **Figs 7B, 7D, 8B and 8D**). Moreover, in the southernmost part of the range of *O*. *rhachophora*, the mean annual temperatures, even at 300 m, are still very high (16–17°C) (**Fig 7D**) and *O*. *kokusai* was not found in collections from these localities. The two species do overlap in a narrow bathymetric zone (ca. 250–300 m depth) in the northern part of their geographic range, which corresponds to the lower and upper limits of the thermal tolerance of each species (**Fig 8C and 8D**). Thus, the bathymetric differentiation is more correlated with mean annual temperatures than water column pressure alone. This example of bathymetric and thermal divergence between two common species in a biogeographically important transition region between tropical, temperate and arctic waters [69–71] could be used in future monitoring of global climate changes or other causes of species range shifts.

### Postlarval characters incorporated into adult morphological patterns

(Figs 20–23)

**Fig 20:**
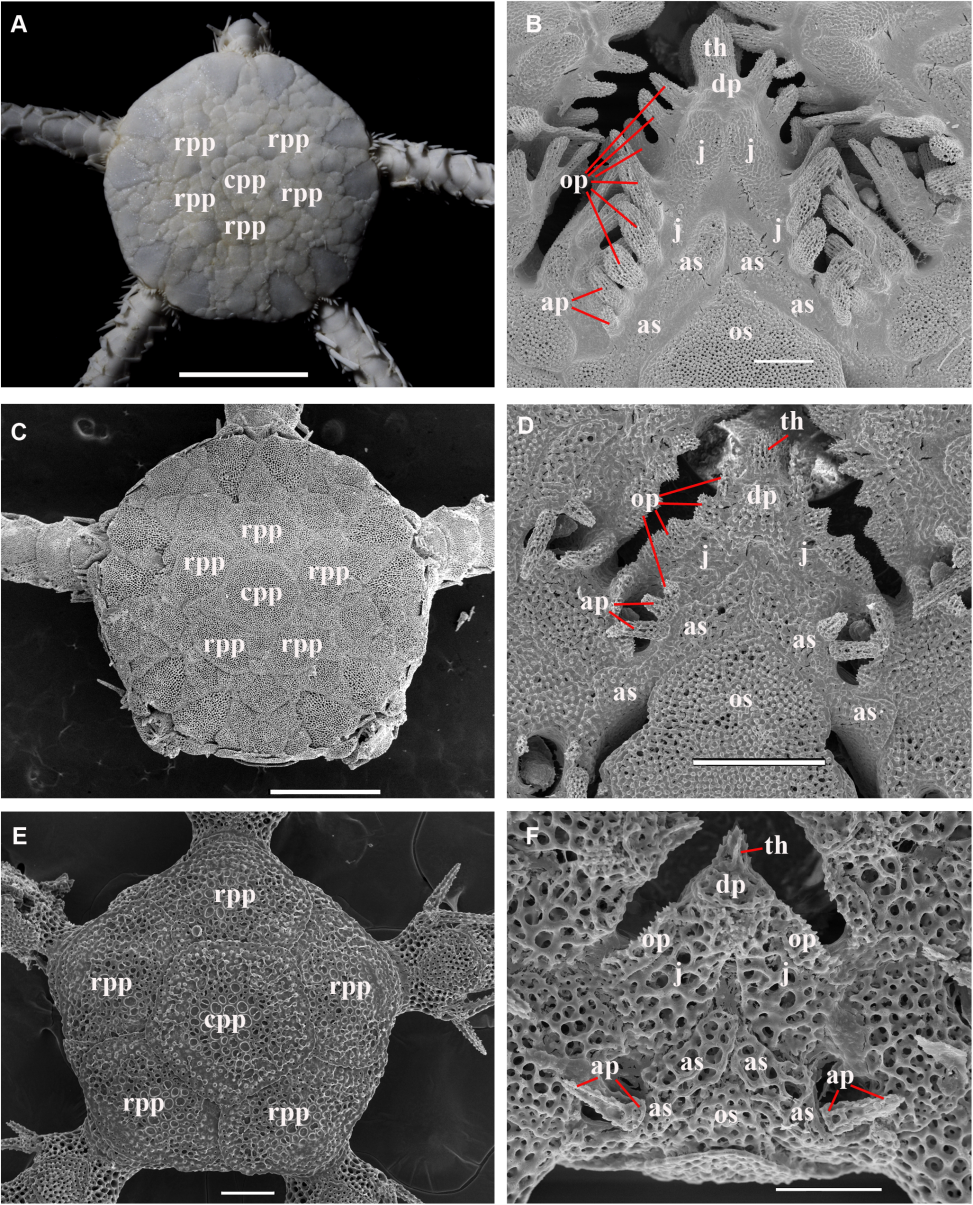
Main stages of the development of *Ophiura leptoctenia* H.L. Clark, 1911 (family Ophiuridae), a series from the locality in Okhotsk Sea (sta. 79). **A, C, E, development of the dorsal disk characters. A,** adult specimen, 10 mm disk diameter, primary plates occupy a restricted area within the disk (ZMMU D–1051); **C,** juvenile specimen, 2 mm disk diameter, primary plates occupy a considerable area of the disk (ZMMU D–1051), SEM; **E,** early postlarval specimen, 1 mm disk diameter, primary plates occupy whole dorsal disk area (ZMMU D–1051), SEM; **B, D, F, development of the ventral oral characters, SEM. B,** adult specimen, 10 mm disk diameter, all oral papillae are spiniform, 2–3 adoral shield papillae; **D,** juvenile specimen, 2 mm disk diameter, both spiniform and block-shaped oral papillae present, 2 pairs of adoral shield papillae; **F,** early postlarval specimen, 1 mm disk diameter, only pair of block-shaped oral papillae present, and pair of adoral shield papillae. ap, adoral shield papillae; as, adoral shields; cpp, central primary plate; dp, dental plate; j, jaws; rpp, radial primary plates; op, oral papillae; os, oral shield; th, teeth. Scales bars, 0.1 mm (E, F), 0.3 mm (B, D), 1 mm (C), 5 mm (A).

**Fig 21:**
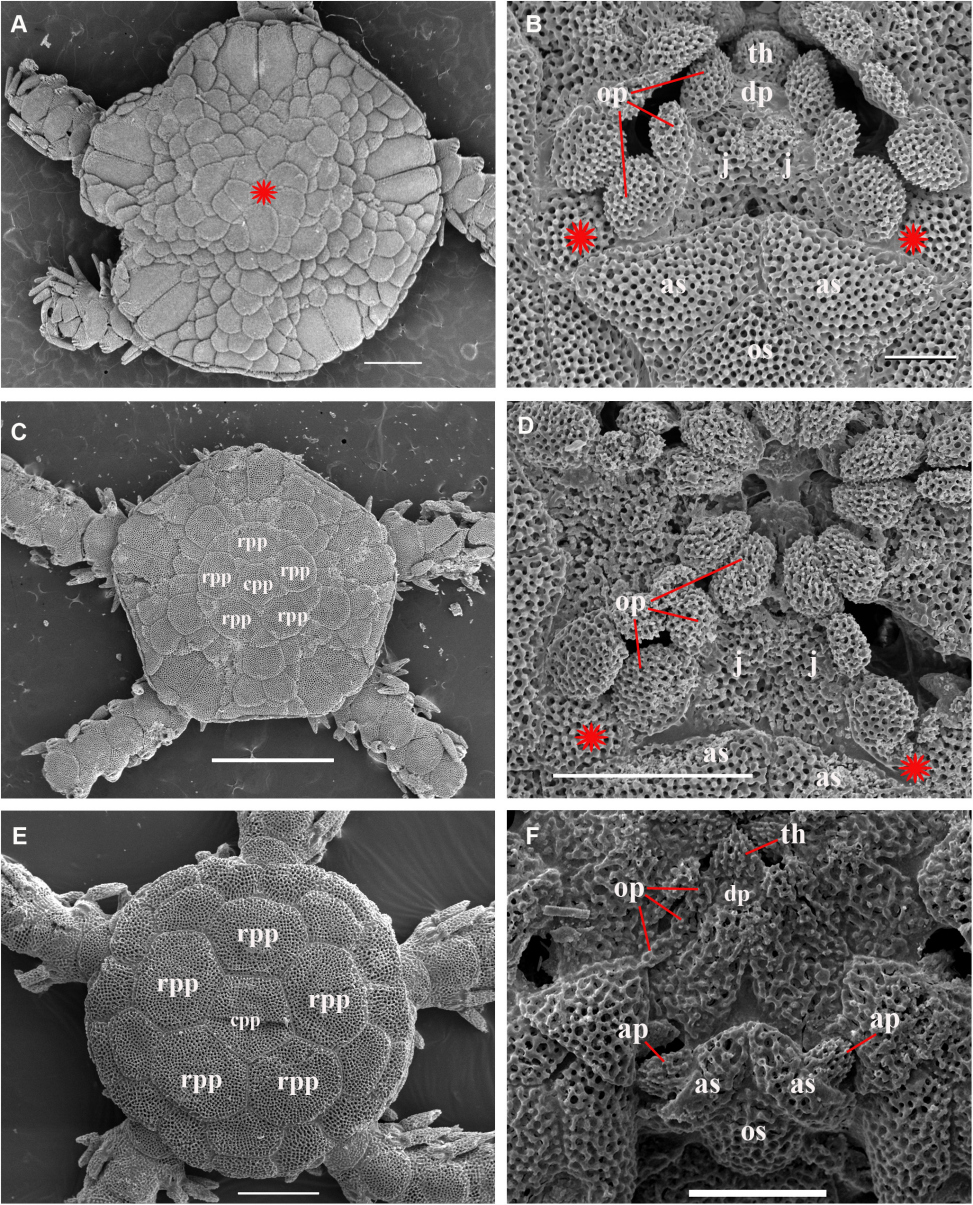
Main stages of the development of *Amphiodia craterodmeta* H.L. Clark, 1911 (family Amphiuridae), a series from the locality in Okhotsk Sea (sta. 37), SEM. **A, C, E, development of the dorsal disk characters. A,** adult specimen, 5 mm disk diameter, primary plates occupy a restricted area of the disk (ZMMU D–1116); **C,** juvenile specimen, 2 mm disk diameter, primary plates occupy a considerable area of the disk (ZMMU D–1116); **E,** early postlarval specimen, 1 mm disk diameter, primary plates occupy major part of the dorsal disk area (ZMMU D–1116); **B, D, F, development of the ventral oral characters. B,** adult specimen, 5 mm disk diameter, all oral papillae are rounded to pointed, no adoral shield papillae; **D,** juvenile specimen, 2 mm disk diameter, all oral papillae are rounded to pointed, no adoral shield papillae; **F,** early postlarval specimen, 1 mm disk diameter, both pointed and block-shaped oral papillae present, pair of adoral shield papillae. ap, adoral shield papillae; as, adoral shields; cpp, central primary plate; dp, dental plate; j, jaws; rpp, radial primary plates; op, oral papillae; os, oral shield; th, teeth; red asterisk on the Fig 20A indicates absence of the definite primary plates rosette, red asterisks on the Fig 20B–20F indicate absence of the adoral shield papllae. Scales bars, 0.1 mm (B, F), 0.3 mm (D, E), 1 mm (A, C).

**Fig 22:**
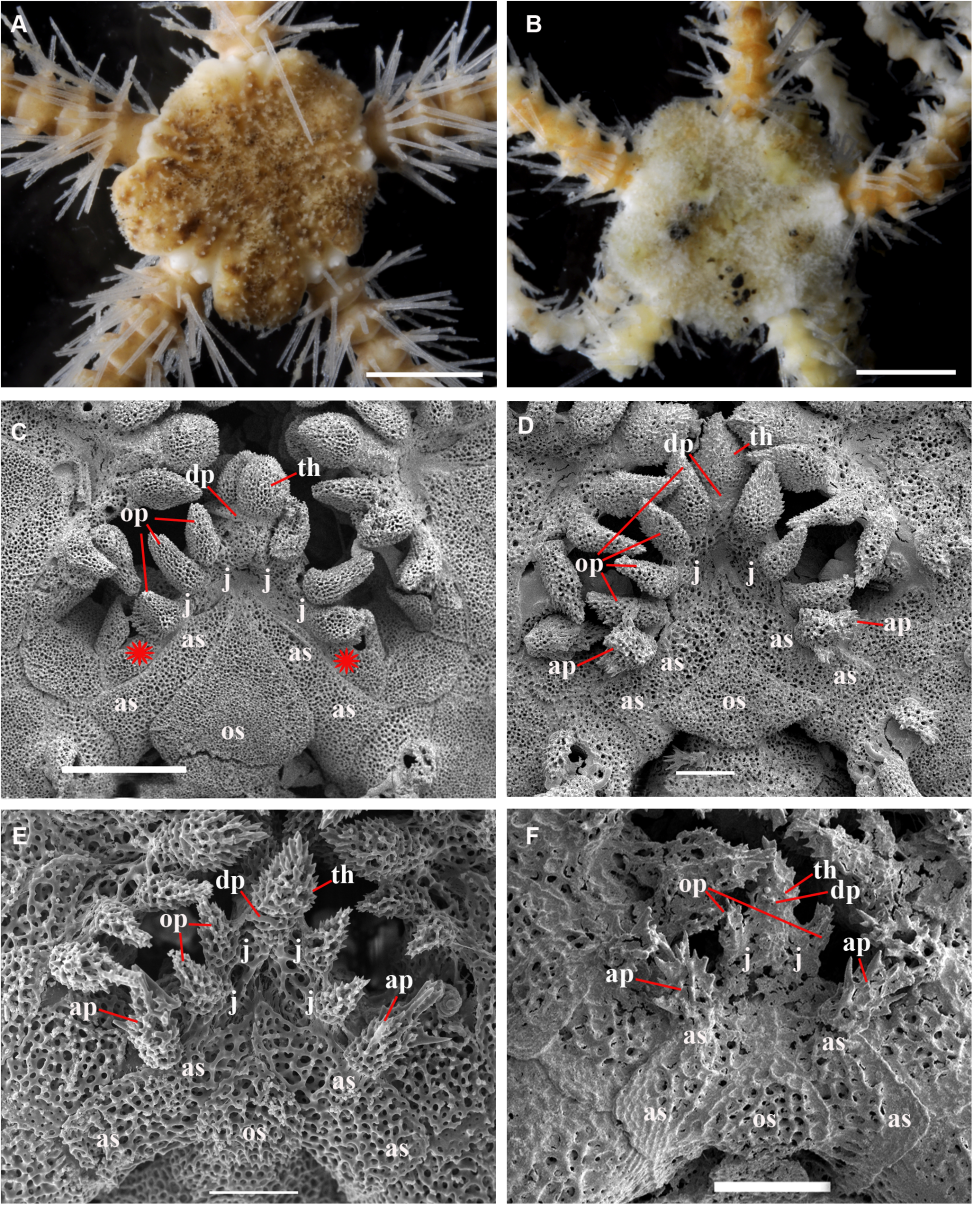
Adult and juvenile characters of *Ophiacantha rhachophora* H.L. Clark, 1911 and *Ophiacantha kokusai* sp.nov. (family Ophiacanthidae). **A, C, E, *Ophiacantha rhachophora*. A,** adult specimen, 4.5 mm disk diameter, dorsal view **(**NSMT E–7609); **C,** oral frame of adult specimen 4.6 mm disk diameter, lobed distal oral papilla is completely migrated from the adoral shield to the jaws (NSMT E–875), SEM; **E,** oral frame of early postlarval specimen, 0.9 mm disk diameter, distal papilla is thorny and attached to the adoral shield (NSMT E–7642), SEM; **B, D, F, *Ophiacantha kokusai* sp. nov. B,** adult specimen, 4 mm disk diameter (NSMT E–7641); **D,** oral frame of adult specimen, 4.2 mm disk diameter, thorny distal “oral” papilla is the adoral shield papilla and is attached to the adoral shield (NSMT E–7589), SEM; **F,** oral frame of early postlarval specimen, 0.5 mm disk diameter, distal papilla is thorny and attached to the adoral shield (NSMT E–7607), SEM. ap, adoral shield papillae; as, adoral shields; dp, dental plate; j, jaws; op, oral papillae; os, oral shield; th, teeth; red asterisks indicate absence of the adoral shield papillae in adult *O*. *rhachophora*. Scales bars, 0.1 mm (F, E), 0.2 mm (D), 0.5 mm (C), 2 mm (A, B).

**Fig 23:**
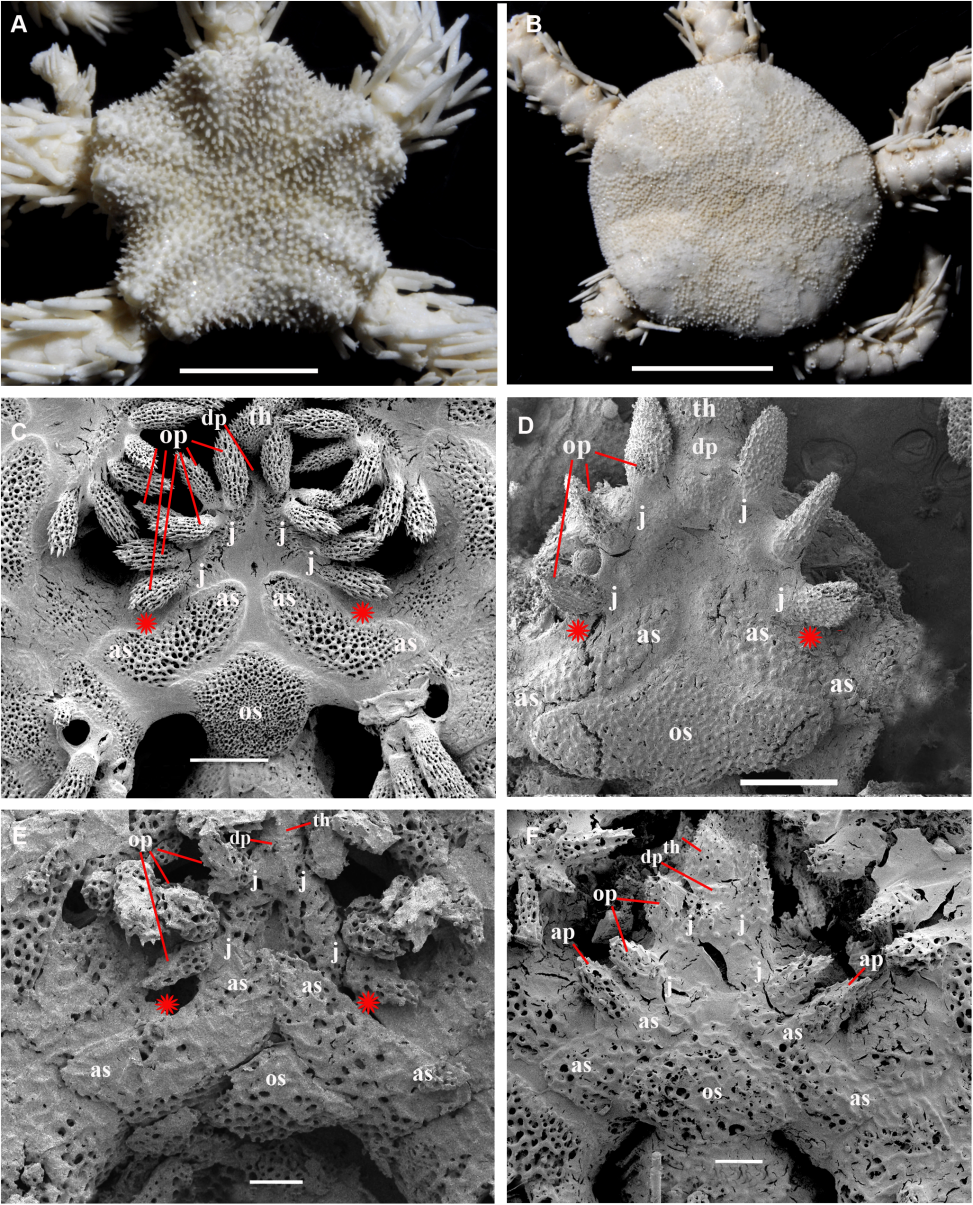
Adult and juvenile characters of *Ophiacantha trachybactra* H.L. Clark, 1911 and *Ophiophthalmus normani* (Lyman, 1879) (family Ophiacanthidae). **A, C, E, *Ophiacantha trachybactra*. A,** adult specimen, 9 mm disk diameter, dorsal view **(**NSMT E–7557); **C,** oral frame of adult specimen 9 mm disk diameter, distal oral papilla is completely detached from the adoral shield (NSMT E–7557), SEM; **E,** oral frame of juvenile specimen, 1.7 mm disk diameter, distal papilla is semi-detached from the adoral shield (NSMT E–7533), SEM; **B, D, F, *Ophiophthalmus normani*. B,** adult specimen, 10 mm disk diameter (NSMT E–2610); **D,** oral frame of the same specimen, distal oral papilla is completely detached from the adoral shield, SEM; **F,** oral frame of juvenile specimen 2 mm disk diameter, distal papilla is the adoral shield spine (ORI KT 90–08), SEM. ap, adoral shield papillae; as, adoral shields; dp, dental plate; gs, genital slit; j, jaws; op, oral papillae; os, oral shield; th, teeth; red asterisks indicate absence of the adoral shield papillae. Scales bars, 0.1 mm (E, F), 0.5 mm (C, D), 4 mm (A), 5 mm (B).

In the majority of the species of the genus *Ophiacantha* (and many other genera of Ophiacanthidae, one of the largest families of ophiuroids) the thorny spine-shaped adoral shield papilla is not part of the adult morphology. However, known ontogenetic data for some species of the genus *Ophiacantha* and other ophiacanthid genera (e.g. adult stages of taxa such as *Ophiolimna* and *Ophiomitrella*) [37, 38] unambiguously demonstrate the presence of the thorny spine-shaped adoral shield papilla at the earlier juvenile stages and its later more proximal migration and further incorporation into the set of oral papillae on the jaws. At the same time, various taxa from other ophiuroid groups, such as in the families Ophiomyxidae and Ophiuridae, may possess non thorny, but still placed on the adoral shield spine-shaped papillae in the adult stages **(Figs 20 and 21)**. The basal position of a group of ex-ophiacanthid genera in relation to other Ophiacanthidae was confirmed with detailed morphological information, novel paleontological data, cladistic and transcriptomic analyses [31, 72, 73]. According to these integrated data, the genus *Ophiacantha* occupies a derived phylogenetic position compared to a basal group of genera such as *Ophioplexa*, *Ophioprium*, *Ophilogimus*, *Ophiomedea*, *Ophiorupta*, which all possess an elaborate set of adoral shield papillae in the adult stage [31] (for distinction between plesiomorphic and pseudo-plesiomorphic conditions see below). Thus the presence of the well-defined adoral shield papillae in an apparently crown species of the genus *Ophiacantha* is of special interest, since it raises questions about the primary or secondary phylogenetic nature of these structures. Small juveniles of *O*. *rhachophora* (1.0–1.8 mm disk diameter) are very similar to *O*. *kokusai* with respect to the shape and placement of the adoral shield papillae **(Fig 22E and 22F)**. During development, the adoral shield papillae in *O*. *rhachophora* migrate proximally, detach from the adoral shields and became the distalmost oral papillae **(Fig 22C)**, whereas in *O*. *kokusai* they remain on the adoral shields **(Fig 22D)**. The placement of the distalmost oral papillae on the jaws in adults of *O*. *rhachophora* and on the adoral shields in adults of *O*. *kokusai* are shown in the present study by the grinding method (**Fig 6B, 6C, 6E and 6F**). This is a clear evidence for ontogenetic migration of the adoral shield papillae to the jaws. Thus, the adult position of the adoral shield papillae in *O*. *kokusai* is almost identical to the placement of these structures in earlier juveniles of *O*. *rhachophora* **(Fig 22E)**.

To further demonstrate the developmental changes when the juvenile adoral shield papillae are incorporated into an adult set of oral papillae, ophiuroid juveniles, subadults and adults belonging to different families have been investigated with special attention to the adoral shield papillae morphology (Table 3) (**Figs 20–23**). These data confirm previous observations [37, 38] on the developmental fate of the adoral shield papillae during postlarval ontogeny. Instructive results were obtained in the course of comparison of the postlarval growth series of two closely related species *O*. *rhachophora* and *O*. *kokusai* (**Fig 22**). The position and shape of the adult adoral shield papillae of *O*. *kokusai* is very similar, not only to the earlier juveniles of the closest species *O*. *rhachophora*, but also to other species of the genus *Ophiacantha* ([38]; present study, Table 3). Because *O*. *kokusai* is similar to the species *O*. *rhachophora*, *O*. *trachybactra* and to some other *Ophiacantha* species, such as *O*. *pentagona*, which also lack adoral shield papillae in the adult stages, and taking into consideration the most recent outline of the ophiacanthid [72] and ophiuroid phylogenies [73], it is unlikely that the adoral shield papillae of the new species are an ancient, plesiomorphic adult feature continuously present from some basal ophiacanthids. Instead, the secondary re-appearence of the adoral shield papillae in *O*. *kokusai* sp. nov. appears to be a heterochronic ontogenetic alteration. Such apparent “pseudo-plesiomorphies” caused by a secondary developmental “push” of the postlarval characters to the adult morphology has misled researches in different metazoans groups (e.g. in amphibians [74] and nudibranch molluscs [75, 76]).

**Table 3:** Changes in placement of the adoral shield papillae during postlarval development among species of three different ophiuroid families.

Taxon	Adoral shield papillae		Disk diameter		Locality	Types (adults)
	Juvenile specimens	Adult specimens	Juveniles (mm)	Adults (mm)		
Fam. Ophiuridae *Ophiura leptoctenia* Clark H.L., 1911 (Fig 20)	Present, spiniform	Present (unchanged placement on adoral shield), spiniform	0.75–0.9	7.0–12.0	Okhotsk Sea	Holotype USNM 25732; 118 paratypes USNM 26994
Fam. Amphiuridae *Amphiodia craterodmeta* Clark H.L., 1911 (Fig 21)	Present, rounded	transformed into distalmost oral papillae	0.9–1.0	5.0–10.0	Okhotsk Sea	Syntype USNM 26019; 10 syntypes USNM 12739
Fam. Ophiacanthidae *Ophiophthalmus normani* (Lyman, 1879) (Fig 23)	Present, spiniform	transformed into distalmost oral papillae	1.8–2.0	8.0–10.0	NW Pacific off central Honshū and Shikoku	BMNH 82.12.203.307
Fam. Ophiacanthidae *Ophiacantha* cf. *pentagona* Koehler, 1897	Present, club-shaped, thorny	transformed into distalmost oral papillae	0.8–2.0	4.0–5.0	NW Pacific off central Honshū	BMNH 98.7.11.12; Syntypes MNHN EcOs 20403
Fam. Ophiacanthidae *Ophiacantha rhachophora* Clark H.L, 1911 (Fig 22)	Present, club-shaped, thorny	transformed into distalmost oral papillae	1.0–2.0	4.0–5.6	NW Pacific along all main Japanese Islands	Holotype USNM 25630
Fam. Ophiacanthidae *Ophiacantha kokusai* sp.nov. (Fig 22)	Present, club-shaped, thorny	Present, club-shaped, thorny	0.8–2.0	4.0–5.1	NW Pacific off central Honshū and Shikoku	Holotype NSMT E–3188

## Conclusions

A newly described species of brittle star *Ophiacantha kokusai* sp. nov. shows signficant bathymetric and thermal differentiation from the closely related species *O*. *rhachophora* H.L. Clark, 1911. The numerically abundant species *O*. *kokusai* demonstrates a number of consistent morphological differences compared with *O*. *rhachophora*, however it remained undetected for over one century in one of the most intensively studied region of Japanese waters. This study demonstrates the importance of incorporating onotogenetic information to taxonomy and phylogenetics. Consistent use of developmental data in taxonomy and phylogenetics on a practical basis brings many potential benefits, including robustly supported reference points in both developmental and evolutionary transformational series and prevents the incorrect phylogenetic assessment of ontogenetically-driven secondary characters. In the current study, we incorporate some elements of an ontogenetic approach to taxonomy, including using a two-fold diagnosis to encompass both juvenile and adult characters. The bathymetric differentiation and environmental preferences of both *O*. *rhachophora* and *O*. *kokusai* make them potentially good indicators for monitoring offshore species range shifts in response to global climate change.

## Supporting Information

S1 Appendix Material examined. (PDF)
