# Supplementary material for: When Ontogeny Matters: A New Japanese Species of Brittle Star Illustrates the Importance of Considering both Adult and Juvenile Characters in Taxonomic Practice
Source: PLoS One. 2015 Oct 28;10(10):e0139463. doi: 10.1371/journal.pone.0139463 (PMC4625035; doi:10.1371/journal.pone.0139463)
Supplement: S1 Appendix — (PDF) [file pone.0139463.s001.pdf]

S1 Appendix. Material examined.

Collecting data	Specimens and museum numbers
***Ophiacantha kokusai* sp. nov.**	**Paratypes**
**Ocean Research Institute, University of Tokyo (*ORI*), R/Vs *Tansei-maru* (KT) and *Hakuho-maru* (KH) surveys in 1973–1996**	
ORI, stn KT-73-06 (D), 10 Jun 1973, SW of Matsuzaki Suruga Bay ,34° 46,1' N 138° 42,4' E – 34° 46,8' N 138° 42,5' E, 306–334 m	15 (NSMT E-7629), 1 (NSMT E-7631)
ORI, stn KT-73-06 (D'), 10 Jun 1973, SW of Matsuzaki Suruga Bay, 34° 46,6' N 138° 42,0' E – 34° 47,5' N 138° 42,1' E, 355–411 m	70 (NSMT E-7634)
ORI, stn KT-74-14 (B4), 23 Sep1974, off Matsuzaki Suruga Bay, 34° 45,8' N 138° 42,5' E – 34° 45,6' N 138° 42,2' E, 312–328 m	10 (NSMT E-7626), 14 (NSMT E-7624)
ORI, stn KT-74-14 (B8), 23 Sep 1974, off Toi Suruga bay, 34° 54,4' N 138° 43,7' E – 34° 53,6' N 138° 43,8' E, 337–355 m	6 (NSMT E-7628)
ORI, stn KT-74-14 (B5-II), 23 Sep 1974, off Matsuzaki Suruga Bay, 34° 46,1' N 138° 40,3' E – 34° 46,1' N 138° 40,0' E, 615–680 m, beam trawl	2 (NSMT E-7509)
ORI, stn KH-74-3 B4-1, 23 Jul 1974 off Tosa Kouchi Pref., 275 m, otter trawl, collector S Irimura	22 (from NSMT E-1122)
ORI, stn KT-76-03 (006), 28 Feb 1976 off Matsuzaki Suruga, Bay 34° 46,10' N 138° 42,65' E – 34° 45,8' N 138° 42,8' E, 262–290 m	3 (NSMT E-7622)
ORI, stn KT-76-03 (BS-I), 02 Mar 1976, Sagami Bank, 478–490 m, beam trawl	1 (NSMT E-7510)
ORI, stn KT-78-11 (OT2), 13 Jul 1978, off Toi Suruga bay, 34° 56,1' N 138° 44,2' E – 34° 55,5' N 138° 44,2' E, 310–320 m	1 (NSMT E-7623)
ORI, stn KH-78-05 (BS 8), 07 Dec 1978 W, off Izu-Oshima, Sagami bay 34° 40,5' N 139° 15,0' E, 415–440 m	100 (NSMT E-7516) 5 (NSMT E-7511)
ORI, stn KT-79-11 (OT 4), 19 Jul1979, off Matsuzaki, Suruga Bay, 34° 46,7' N 138° 41,4' E – 34° 47,7' N 138° 42,7' E, 405–457 m	1 (NSMT E-7635) 9 (NSMT E-7632)
ORI, stn KT-79-11 (OT-3), 19 Jul 1979, W off Matsuzaki Suruga Bay, 34° 46,7' N 138° 42,5' E – 34° 45,3' N 138° 42,4' E, 295–310 m, otter trawl	3 (NSMT E-7512)
ORI, stn KT-80-11 (OT-2), 08 Jul 1980, W of Matsuzaki Suruga Bay, 34° 44,5' N 138° 42,0' E – 34° 44,5' N 138° 41,6' E, 370–372 m, beam trawl	57 (NSMT E-7519)
ORI, stn KT-83-18 (H-4), 11 Nov 1983, off Hakachi-zaki, Cape Izu Pen-	19 (NSMT E-1734)

Peninsula, Suruga Bay, Shizuoka Pref- 34° 40,8' N 138° 41,5' E, 367–375 m, otter trawl, collector S Irimura	
ORI, stn KT-84-12 (6) 30 Aug1984, SW off Kushimoto, 33° 24,531' N 135° 42,403' E – 33° 24,441' N 135° 43,639' E, 314–335 m, beam trawl	1 (NSMT E-7520)
ORI, stn KT-86-06 (KN 4), 27 May1986, Kumano trough, 34° 05,9' N 136° 40,8' E – 34° 05,9' N 136° 38,7' E, 422–425 m, beam trawl	1,125 (NSMT E-7630), 27 (NSMT E-7521)
ORI, stn KT-87-19 (TW 2) 08 Dec1987, Takase west, 221–244 m	1 (NSMT E-7627)
ORI stn KT-87-19 (OM 4), 10 Dec 1987, Omurodashi Sagaminada, OR Sagami Sea, 409–437 m	7 (NSMT E-7508), 901 (NSMT E-7638)
ORI, stn KT-76-16 (C-1), 17 Feb1992, between Yakushima Id and Tanegashima Id- Kagoshima Pref- 30° 4,6' N 130° 55,7' E, sand, 402–410 m, beam trawl	2 (from NSMT E-3137)
ORI, stn KT-92-11 (OM 4), 30 Jul1992, Omurodashi Sagaminada, 397–413 m, ORI biological dredge	77 (NSMT E-7513)
ORI, stn KT-95-05 (TB 14), 26 Apr1995, SE off Taitozaki Bōsō Peninsula, 35° 09,31' N 140° 48,57' E – 35° 09,60' N 140° 49,40' E, 311–325 m	3 (NSMT E-7625)
ORI, stn KT-95-17 (EN1–5), 10 Dec 1995, W of Omaezaki Enshunada 34° 22,786' N 137° 59,999' E – 34° 24,003' N 137° 59,973' E, 463–491 m	1 (NSMT E-7633)
ORI, stn KT-95-17 (EN1–7), 10 Dec 1995, W of Omaezaki Enshunada 34° 23,194' N 137° 59,998' E – 34° 24,454' N 138° 00,457' E, 444–478 m	1 (NSMT E-7636)
ORI, stn KT-95-17 (EN1-3), 10 Dec1995, 1st Tenryu Knoll Enshunada 34° 22,865' N 138° 00,057' E – 34° 24,018' N 138° 00,108' E, 457–489 m beam trawl	1 (NSMT E-7507)
ORI, stn KT-95-17 (KN1–2), 12 Dec 1995, Kumano Basin 34° 06,960' N 136° 39,517' E – 34° 05,992' N 136° 38,747' E, 420–425 m	49 (NSMT E-7637) 4 (NSMT E-7514)
ORI, stn KT-98-14 (2), 29 Apr 1998, W off Izu-Oshima, 257–264 m, beam trawl	2 NSMT E-7517)
	6 (NSMT E-7515), 28 (NSMT E-7518)-
**Tosa Bay surveys 1997–2000**	
stn K97-5-150, 14 May1997, 33° 15,0' N, 133° 36,5' E – 33°14,6' N, 133° 362' E, 151–155 m	5 (NSMT E-7565)
stn K97-11-300, 14 May 1997, 33° 10,6' N, 133° 34,4' E – 33° 10,3' N,	1 (NSMT E-7576), .3

133° 33,9' E, 300–302 m	(NSMT E-7567), 34 (NSMT E-7569), 3 (NSMT E-7568), 29 (NSMT E-7566),
stn K98-9-300, 11 Sep 1998, 33° 10,6' N, 133° 34,4' E – 33° 10,4' N, 133° 33,7' E, 284–302 m	11 (NSMT E-7569B),
stn K99-9-400, 27 Sep 1999, 33° 12,5'N, 133° 38.6' E – 33°12,2' N, 133° 38,0' E, 364–382 m	51 (NSMT E-7564)
stn K00-5-300, 09 May 2000, 33° 14,9' N, 133° 40,3' E – 33°15,3' N, 133° 41,0' E, 298 m	1 (NSMT E-7573)
stn K00-5-400, 09 May 2000, 33° 13,2' N, 133° 40,1' E – 13,6' N, 133° 40,6' E, 397 m.	9 (NSMT E-7574)
stn K00-8-400, 24 Aug 2000, 33° 13,5' N, 133° 41,6' E – 33° 12,8' N, 133° 41,4' E, 440–460 m	1 (NSMT E-7575)
**Sagami Bay surveys 2000–2003**	
R/V *Hajime-maru*, stn 2, 23 Dec 2000, Eastern Sagami Bay, 35° 13.251' N 139° 26.342' E – 35° 13.178' N 139° 26.374' E, 370 m, G: gill net, C: cage, D: dredge	1 (NSMT E-7577)
R/V *Rinkai-maru*, stn 1, 20 Feb 2001, Eastern Sagami Bay, 35° 9,26' N 139° 30,99' E – 35° 9,22' N 139° 31,05' E, 573–610 m, D	1 (NSMT E-7578)
R/V *Rinkai-maru*, stn 2 , 20 Feb 2001, Eastern Sagami Bay, 35° 9,70' N 139° 31,09' E – 35° 9,52' N 139° 31,123' E, 327–418 m, D	3 (NSMT E-7579)
R/V *Rinkai-maru*, stn 2 , 20 Feb 2001, Eastern Sagami Bay, 35° 9,26' N 139° 30,99' E – 35° 9,22' N 139° 31,05' E, 573–610 m, D	2 [159-7]
Fishing boat *Daisan idoinkyo-maru*, lobster's station 1, 05 Mar 2002, Uragasuido, 35° 4,56' N 139° 40,04' E – 35° 4,04' N 139° 39,63' E, ca. 300 m, C	1 (NSMT E-7580), 1 (NSMT E-7581)
R/V *Shinyō-maru*, stn 4, 22 May 2002, off Misaki, 336–303 m, D.	6 (NSMT E-7597) (SEM stub # 15)
R/V *Shinyō-maru*, stn 5, 22 Oct 2002, off Misaki, 35° 7' N 139° 33,7' E – 35° 6,6' N 139° 33,8' E, 313–332 m, D	1 (NSMT E-7594),<br>2 (NSMT E-7595)
R/V *Shinyō-maru*, stn 27, 24 Oct 2002, off Oshima, 34° 38,4' N 139° 17,83' E – 34° 39,24' N 139° 17,64' E, 356–348 m	6 (NSMT E-7587)
R/V *Shinyō-maru*, stn 28, 24 Oct 2002, off Izu-Oshima, 34° 40' N 139° 17,5' E – 34° 39,9' N 139° 17,9' E, 327–333 m, D	4 (NSMT E-7583), 10 (NSMT E-7584), 2 (NSMT

	E-7585), 12 (NSMT E-7586), 2 (NSMT E-7588) (SEM stub # 1), 2 (NSMT E-7588), 5 (NSMT E-7589)
R/V *Shinyō-maru*, stn 27, 24 Oct 2002, off Izu-Oshima, 38,4° N 139° 17,83' E – 34° 39,24' N 139° 17,64' E, 356–348 m, D	1 (NSMT E-7591)
R/V *Shinyō-maru*, stn 29, 24 Oct 2002, off Izu-Oshima, 34° 40,2 N 139° 18,6' E – 34° 40,4' N 139° 18,4' E, 307–289 m, D	1 (NSMT E-7592)
R/V *Shinyō-maru*, stn 38, 24 Oct 2002, off Izu-Oshima, 34° 44,54' N 139° 18,59' E – 34° 44,36' N 139° 18,44' E, 346–343 m, D	1 (NSMT E-7593)
R/V *Shinyō-maru*, stn 42, 25 Oct 2002, off SW Suzaki, 34° 51,37' N 139° 38,94' E – 34° 50,79' N 139° 39,28' E, 452–381 m, D	2 (NSMT E-7596) (SEM stub # 3)
R/V *Shinyō-maru*, stn 9, 18 Oct 2003, off SW Suzaki, 34° 55,5' N 139° 40,2' E – 34° 55,4' N 139° 40,5' E, 375–275 m, D	5 (NSMT E-7601), 1 (NSMT E-7603), 1 (NSMT E-7606)
R/V *Shinyō-maru*, stn 10, 18 Oct 2003, off SW Suzaki, 34° 54,8' N 139° 39,7' E – 34° 54,8' N 139° 39,9' E, 348–312 m, D	2 (NSMT E-7641), 1 (NSMT E-7604), 1 (NSMT E-7605)
R/V *Shinyō-maru*, stn 11, 18 Oct 2003, off SW Suzaki, 34° 54,2' N 139° 39,9' E – 34° 54,3' N 139° 39,3' E, 315–365 m, D	6 (NSMT E-7598), 3 (NSMT E-7599), 13 (NSMT E-7639), 6 (NSMT E-7607)
**Kii Peninsula and Takase Bay surveys**	
T/S *Seisui-maru*, 15 Jun 1991, off Owase, Kii Peninsula, Wakayama Pref., sand, 237–333 m, benthos net, collector M. Saba	1 (NSMT E-3178)
T/S *Seisui-maru*, sand, 16 Nov 990, off Owase Trough, Kii Peninsula, Wakayama Pref., 300–500 m, basket, collector M. Saba	4 (NSMT E-3185)
T/S *Seisui-maru*, sand, 06 Nov 1990, off Adawa, Kii Peninsula, Wakayama Pref., 378–617 m, basket, collector M. Saba	7 (NSMT E-3195)
T/S *Seisui-maru*, sand, 30 Mar 1990, Koyo Trough, Kii Peninsula, Wakayama Pref., 33° 55,6' N 136° 19,1' E, 277–427 m, benthos net, collector M. Saba	11 (NSMT E-3206)
R/V *Soyo-maru*, stn D-72, 27 May 1986, Takase Bank, 34° 23,6' N 139° 11,8' E , 340 m, beam trawl.	4 (NSMT E-3235),

Ophiacantha rhachophora **H.L. Clark, 1911**	**Other material**
Izu-Oshima, 19 Nov 1963, 56 m.	30 (NSMT E-7563),
R/V *Soyo-maru*, 68B st. B-1, sand, 30 Nov 1968, off Chikura, Bōsō Peninsula, Chiba Pref., 34° 55,1' N 140° 2,7' E, 270–305 m, beam trawl, collector T. Okutani.	3 (NSMT E-2633, splitted)
St. 12, 07 Dec 1968, Nakanose, Sagami Bay, 50 m, beam trawl, collector S. Gamo.	4 (NSMT E-3905, splitted),
R/V *Tansei-maru*, stn KT-69-12, T-2, 13 Jul 1969, off Matsuzaki, Izu Peninsula, Suruga Bay, Shizuoka Pref., 34° 44 ' N 138° 42,8' E, 80 m, dredge, collector S. Irimura.	4 (NSMT E-871),
R/V *Tansei-maru*, stn KT-69-12, T-3, 13 Jul 1969, E off Irou-zaki Cape, Izu Peninsula, Shizuoka Pref., 34° 41,8 ' N 138° 43,8' E, 86 m, dredge, collector S. Irimura.	2 (NSMT E-875; SEM stub # 5),
R/V *Tansei-maru*, stn KT-69-12, St.T-13, 14 Jul 1969, off Shimoda, Izu Peninsula, Shizuoka Pref., 34° 37,1 ' N 138° 58,8' E, 138–155 m, beam trawl, collector S. Irimura.	4 (NSMT E-884; SEM stubs # 8, 9),
R/V *Tansei-maru*, tn KT-69-12, 14 Jul 1969, off Shimoda, Izu Peninsula, Shizuoka Pref. , 34° 37,5 ' N 138° 37,5' E, 54–72 m, beam trawl, collector S. Irimura.	3 (NSMT E-887),
R/V *Hakuhō-maru* , stn KH-74-3, B4-1, 23 Jul 1974, off Tosa, Kouchi Pref., 275 m, otter trawl, collector S. Irimura	28 (NSMT E-1122)
R/V *Hakuhō-maru*, stn KH-74-3, B4-2, 23 Jul 1974, off Tosa, Kouchi Pref., 263 m, otter trawl, collector S. Irimura	1 (NSMT E-1138)
ORI, stn KT-75-15 (02), 24 Nov 1975, off Toi, Suruga bay, 34° 55,83' N 138° 44,85' E – 34° 56,62' N 138° 45,0' E, 192–205 m	2 (NSMT E-7559), 6 (NSMT E-7558)
R/V *Tansei-maru*, stn KT-76-16, C-1, sand, 25 Sep 1976, off Bōsō Peninsula, Chiba Pref., 34° 25,7' N 137° 58,5' E, 150 m, otter trawl, collector S. Irimura	118 (NSMT E-1540)
ORI, stn KT-78-11 (OT-1), 13 Jul 1978, off Toi, Suruga bay, 34° 56,1' N 138° 44,8' E – 34° 55,6' N 138° 44,9' E, 200–205 m	2 (NSMT E-7560)
ORI, stn KT-79-11 (OT-6), 19 Jul 1979, W off Matsuzaki, Suruga Bay, 34° 46,5' N 138° 43,0' E – 34° 44,8' N 138° 42,6' E, 215–277 m, otter trawl.	1 (NSMT E-7561)
R/V *Seishin Maru*, stn DG94-8, 26 Oct 1994, Suruga Bay, 150–200 m.	1 (NSMT E-4279)

ORI, 13 Jul 1996, Sagami Bay, SE of Iritahama, 34° 36,54' N 138° 59,514' E 34° 36,566' N 138° 59,745' E, 210–218 m, D	11 (NSMT E-7614, SEM stub # 4)
ORI, stn KT-98-14 (2), 29 Apr 1998, W off Izu, Oshima, 257–264 m	1 (NSMT E-7562),
R/V *Toyoshio-maru*, stn To-01 St.8, 28 May 2001, Amami Oshima NW, 28° 29' N 129° 31,5' E, 172 m, ORI Biological dredge	1 (NSMT E-7620)
R/V *Toyoshio-maru*, stn. TO-02-05 St. 9; 26 May 2002, Aguni East, 26° 30,72' N 127° 25,99' E – 26° 30,31' N 127° 26,13' E, 439-487 m, beam trawl, collector Fujita T., Torigoe T.	1 (NSMT E-7619)
R/V *Toyoshio-maru*, stn TO-02-05 St. 12, 27 May 2002, Iejima North, 26° 48,23' N 127° 52,18' E – 26° 48,28' N 127° 52,8' E, 337–340 m, beam trawl	3 (NSMT E-7621)
stn 1, 24 Oct 2002, off Oshima, 34° 40,65' N 139° 19,29' E – 34° 40,75' N 139° 19,02' E, 228–252 m, D	1 (NSMT E-7608)
R/V *Suzaki nisei*, stn 1, 17 Mar 2005, off Shimoda, 34° 36,324' N 138° 59,144' E – 34° 36,562' N 138° 59,15' E, 193–210 m, D	1 (NSMT E-7613), 1 (NSMT E-7611), 1 (NSMT E-7610), 1 (NSMT E-7612, SEM stub # 30)
Ophiacantha trachybactra **H.L. Clark, 1911**	**Other material**
ORI, stn KH-67-02 (4), 12 Aug1967, E off Kesennuma, 1970–1990 m, beam trawl	1 (NSMT E-7549)
ORI, stn KH-67-05 (1), 03 Dec 1967, E off Nakaminato, 1690 m, beam trawl	3 (NSMT E-7554)
ORI , stn B2, off Miyake-jima Id., Soyo, 25 Jul 1975, 650–690 m, collector T. Okutani, beam trawl	1 (NSMT E-2786)
ORI, stn KH-81-04 (8), 25 Jul 1981, E off Kesennuma, 1970–1990 m, beam trawl	1 (NSMT E-7542)
ORI, stn KT-81-19 (F5), 01 Dec 1981, E off Soma, Fukushima, 538–561 m, beam trawl	4 (NSMT E-7536)
ORI, stn KH-81-04 (8), 25 Jul 1981, E off Kinkasan, Sanriku, 1976–1987 m, beam trawl	8 (NSMT E-7529),<br>2 (NSMT E-7543)
ORI, stn KT-84-09 (SR 6), 10 Jul 1984, E off Otsuchi, Sanriku, 926–950 m, beam trawl	8 (NSMT E-7534)
R/V *Tansei-maru*, stn KT-84-9 (SR 8-2), 11 Jul 1984, off Oodzuchi,	2 (NSMT E-1904)

(Iwate Pref.), 38° 57,3' N 142° 56,9' E, 1430–1440 m, collector E. Tsuchida, trawl	
ORI, stn KT-85-11 (SR-16), 12 Aug 1985, SE off Otsuchi, Sanriku, 1225–1249 m, beam trawl	2 (NSMT E-7538)
ORI, stn KT-87-05 (SR-33), 21 May 1987, E off Otsuchi, Sanriku, 824–840 m, beam trawl	4 (NSMT E-7525)
ORI, stn KT-87-05 (SR-35-1), 21 May 1987, SE off Otsuchi, Sanriku, 1237–1255 m, beam trawl	1 (NSMT E-7546)
R/V *Tansei-maru*, stn KT 87-5 (SR 35), 21 May 1987, off Oodzuchi, (Iwate Pref.), 39° 0,9' N 142° 37' E, 1237–1255 m, collector S.Irimura, trawl	1 (NSMT E-2000)
ORI, stn KT-87-05 (SR-35F), 21 May 1987, SE off Otsuchi, Sanriku,1301–1308 m, beam trawl	1 (NSMT E-7540)
ORI, stn KT-87-05 (KS-1), 19 May 1987, E off Kashima, 1607–1727 m, beam trawl	2 (NSMT E-7553)
ORI, stn KT-87-05 (SR 34A), 20 May 1987, SE off Otsuchi, Sanriku, 1038–1055 m, beam trawl	1 (NSMT E-7541)
ORI, stn KT-88-07 (SR 51), 24 May 1988, E off Otsuchi, Sanriku, 1205–1213 m, beam trawl	1 (NSMT E-7530)
ORI, stn KT-89-14 (TB-12), 15 Sep 1989, E off Bōsō Peninsula, 1361–1390 m, beam trawl	2 (NSMT E-7555)
ORI, stn KT-89-14 (SR-117), 17 Sep 1989, E off Otsuchi, Sanriku, 531–543 m, beam trawl	1 (NSMT E-7552)
ORI, stn KT-89-14 (SR-118), 16 Sep 1989, SE off Otsuchi, Sanriku, 979–990 m, beam trawl	1 (NSMT E-7522)
ORI, stn KT-89-14 (SR-120), 18 Sep 1989, SE off Otsuchi, Sanriku, 1715–1773 m, beam trawl	1 (NSMT E-7551)
ORI, stn KT-87-05 (KS-1), 19 May 1987, E off Kashima, Kasimanada, 1647–1727 m, beam trawl	2 (NSMT E-7526)
ORI, stn KT-90-07 (SR 102), 27 May 1990, SE off Otsuchi, Sanriku, 975–988 m, beam trawl	12 (NSMT E-7556)
ORI, stn KT-90-08 (KR 09), 14 Jun 1990, SE off Kushiro, 992–1042 m, beam trawl	1 (NSMT E-7548)
ORI, stn KT-90-08 (St. KR 11), 15 Jun 1990, S off Kashiro, 1475–1498	4 (NSMT E-7544)

m, beam trawl	
ORI, stn KT-90-08 (SR 126), 17 Jun 1990, E off Miyako, 1376–1450 m, beam trawl	5 (NSMT E-7550)
ORI, stn KT-85-11 (SR 17), 12 Aug 1990, SE off Otsuchi, Sanriku, 1642–1659 m, beam trawl	3 (NSMT E-7533, SEM stub #51)
ORI, stn KT-91-06 (12), 16 Nov 1991, E off Miyako, Sanriku, 1806–1830 m, beam trawl	1 (NSMT E-7524)
ORI, stn KT-93-15 (04), 13 Nov 1993, E off Otsuchi, Sanriku, 1132–1170 m, beam trawl	2 (NSMT E-7532)
ORI, stn KT-93-15 (T 10), 16 Nov 1993, S off Akkeshi, Hokkaido, 1938–1969 m, beam trawl	1 (NSMT E-7547)
ORI, stn KT-93-15 (M 2), 18 Nov 1993, E off Miyako, Sanriku, 541–553 m, beam trawl	1 (NSMT E-7545)
ORI, stn KT-93-15 (M 3), 18 Nov 1993, E off Kuji, Sanriku, 1005–1016 m, beam trawl	14 (NSMT E-7531), 4 (NSMT E-7557, SEM stubs ## 43–49)
ORI, stn KT-95-05 (TB 17), 24 Apr 1995, SE off Taitosaki, Bōsō Peninsula, 1351–1454 m, beam trawl	11 (NSMT E-7535), 1 (NSMT E-7539)
R/V *Wakataka-maru*, stn 27, 19 May 1995, off Oodzuchi, (Iwate Pref.), 38° 0' N 142° 13,6' E, 1195–1201 m, trawl	2 (NSMT E-4725)
ORI, stn KT-98-14 (16-2), NE off Miyake-jima Id., 34° 17,548' N 140° 02,584' E – 34° 17,364' N 140° 04,34' E, 02 Sept 1998, 1575–1620 m, beam trawl	1 (NSMT E-7537)
ORI, stn KT-99-06 (St. 14), 18 May 1999, SE off Kamogaura, Bōsō Peninsula, 1697–1763 m, beam trawl	1 (NSMT E-7523, SEM stub # 39)
ORI, stn KT-00-05 (05), 16 May 2000, SE off Kamogawa, Bōsō Peninsula, 580–1702 m, beam trawl	1 (NSMT E-7616)
ORI, stn KT-00-05 (St. 03), 20 May 2000, SE off Kamogawa, Bōsō Peninsula, 692–696 m, beam trawl	1 (NSMT E-7528)
R/V *Rinkai-maru*, stn 1, 20 Feb 2001, Eastern Sagami Bay, 35° 9,26' N 139° 30,99' E – 35° 9,22' N 139° 31,05' E, 573–610 m, D	1 (NSMT E-7618)
Fishing boat *Daisan idoinkyo-maru*, stn 1, 14 Mar 2001, Uragasuidō, 35° 3,56' N ¬139° 44,44' E – 35° 3,82' N 139° 43, 502' E, 500 m, C	1 (NSMT 122-3)
Fishing boat *Daisanido inkyo-maru*, stn 3, 26 Mar 2003, off Tomiura, 35°	1 (NSMT 1027-4-4)

3,44' N 139° 46,176' E – 35° 2,818' N 139° 45,557' E, 600–640 m, C	
R/V *Shinyō-maru*, stn 6, 18 Oct 2003, off NE Suzaki, 35° 0,7' N 139° 39,8' E – 35° 0,5' N 139° 40' E, 324–530 m, D	1 (NSMT 1251-2)
ORI, stn KT-03-17 (TS 6-5), 18 Nov 2003, E off Taitosaki, Bōsō Peninsula, 971–1011 m, beam trawl	1 (NSMT E-7617)
